# The *Plasmodium berghei* Ca^2+^/H^+^ Exchanger, PbCAX, Is Essential for Tolerance to Environmental Ca^2+^ during Sexual Development

**DOI:** 10.1371/journal.ppat.1003191

**Published:** 2013-02-28

**Authors:** David S. Guttery, Jon K. Pittman, Karine Frénal, Benoit Poulin, Leon R. McFarlane, Ksenija Slavic, Sally P. Wheatley, Dominique Soldati-Favre, Sanjeev Krishna, Rita Tewari, Henry M. Staines

**Affiliations:** 1 Centre for Genetics and Genomics, School of Biology, Queen's Medical Centre, University of Nottingham, Nottingham, United Kingdom; 2 Faculty of Life Sciences, University of Manchester, Manchester, United Kingdom; 3 Department of Microbiology and Molecular Medicine, Centre Medical Universitaire, University of Geneva, Geneva, Switzerland; 4 Centre for Infection and Immunity, Division of Clinical Sciences, St. George's, University of London, London, United Kingdom; 5 Instituto de Medicina Molecular, Faculdade de Medicina, Universidade de Lisboa, Av. Prof. Egas Moniz, Lisbon, Portugal; 6 School of Biomedical Sciences, Queen's Medical Centre, University of Nottingham, Nottingham, United Kingdom; Faculdade de Medicina da Universidade de Lisboa, Portugal

## Abstract

Ca^2+^ contributes to a myriad of important cellular processes in all organisms, including the apicomplexans, *Plasmodium* and *Toxoplasma*. Due to its varied and essential roles, free Ca^2+^ is tightly regulated by complex mechanisms. These mechanisms are therefore of interest as putative drug targets. One pathway in Ca^2+^ homeostatic control in apicomplexans uses a Ca^2+^/H^+^ exchanger (a member of the cation exchanger family, CAX). The *P. falciparum* CAX (PfCAX) has recently been characterised in asexual blood stage parasites. To determine the physiological importance of apicomplexan CAXs, tagging and knock-out strategies were undertaken in the genetically tractable *T. gondii* and *P. berghei* parasites. In addition, a yeast heterologous expression system was used to study the function of apicomplexan CAXs. Tagging of *T. gondii* and *P. berghei* CAXs (TgCAX and PbCAX) under control of their endogenous promoters could not demonstrate measureable expression of either CAX in tachyzoites and asexual blood stages, respectively. These results were consistent with the ability of parasites to tolerate knock-outs of the genes for TgCAX and PbCAX at these developmental stages. In contrast, PbCAX expression was detectable during sexual stages of development in female gametocytes/gametes, zygotes and ookinetes, where it was dispersed in membranous networks within the cytosol (with minimal mitochondrial localisation). Furthermore, genetically disrupted parasites failed to develop further from “round” form zygotes, suggesting that PbCAX is essential for ookinete development and differentiation. This impeded phenotype could be rescued by removal of extracellular Ca^2+^. Therefore, PbCAX provides a mechanism for free living parasites to multiply within the ionic microenvironment of the mosquito midgut. Ca^2+^ homeostasis mediated by PbCAX is critical and suggests plasmodial CAXs may be targeted in approaches designed to block parasite transmission.

## Author Summary

Calcium is vital to all living organisms. It is used within cells to regulate many essential processes and, because of this, its cellular concentration is tightly controlled. To change cellular calcium levels, cells use calcium transport proteins. These proteins can alter calcium concentration by moving calcium into or out of the cell or specialised calcium storage compartments within the cell. We know little about how single-celled apicomplexan parasites, including *Plasmodium* (the causal agent of malaria) and *Toxoplasma* (the causal agent of toxoplasmosis), regulate their calcium levels. Here, we have demonstrated that removing apicomplexan genes for a protein that exchanges calcium for protons across membranes (a Ca^2+^/H^+^ exchanger) and a member of the cation exchanger (CAX) family, does not affect the survival of parasites during those stages when they live within host cells. It is, however, lethal for the mouse malaria *P. berghei* when the parasite is free living within its mosquito vector. When we removed calcium from around the parasites at this stage they were able to develop normally, suggesting that the protein provides a mechanism for the parasite to tolerate environmental calcium. Learning how this calcium transport protein impacts on the development of apicomplexan parasites may lead to the development of novel anti-parasitic interventions.

## Introduction

Free Ca^2+^ is essential for signalling in all cell types and plays a central role in many processes during the complex life cycles of apicomplexan parasites (*e.g. Plasmodium* and *Toxoplasma*), including secretion of adhesins, motility, cellular invasion and egress, and intracellular development [1]–[3]. Proteins that interact with Ca^2+^ are therefore important to identify as novel drug targets and can provide fundamental insights into the biology of parasites when they are functionally characterised [4].

Our current understanding of Ca^2+^ homeostatic control in apicomplexan parasites is limited. To control cytosolic free Ca^2+^ concentrations, eukaryotic cells use a range of Ca^2+^ binding proteins, Ca^2+^ diffusive “leak” pathways and active Ca^2+^ transporters. For example, several apicomplexan P-type Ca^2+^ ATPases have been characterised, such as the sarco(endo)plasmic reticulum Ca^2+^ ATPases (SERCAs) of *P. falciparum*, PfATP6, and *T. gondii*, TgSERCA [5], [6]. *Plasmodium* P-type ATPases are already being investigated as new or existing drug targets. More recently, an initial characterisation of the *P. falciparum* Ca^2+^/H^+^ exchanger (PfCAX, also termed the Ca^2+^/H^+^ antiporter, PfCHA) has been undertaken [7].

PfCAX and other apicomplexan orthologues belong to the Ca^2+^/cation antiporter (CaCA) superfamily and members have been identified across the biological Kingdoms including some lower vertebrates, although not in more complex metazoa including mammals [8], [9]. CAX genes are classified into 3 subfamilies. Type II CAXs are found in fungi, *Dictyostelium*, and lower vertebrates and Type III CAXs are found in bacteria, while Type I CAXs include bacterial, fungal, plant and protozoan CAXs. Type I CAXs are divided into 8 subgroups (A to H) and protozoa are classified into the Type I-C phylogenetic group. The first member of this group to be characterised functionally was a Ca^2+^/H^+^ exchanger (CrCAX1) from the unicellular green alga *Chlamydomonas reinhardtii* [10].

Functional characterisation of CAX proteins (mainly from plants and fungi) has shown that these H^+^ coupled exchangers all transport Ca^2+^, with some being highly specific for Ca^2+^, whilst others mediate the transport of a broad range of additional divalent cations or, in some cases, transport additional monovalent cations [11]–[13]. Their primary role in plants and fungi is to enable tolerance to high extracellular Ca^2+^ concentrations, by internal sequestration of Ca^2+^ into acidic organelles when cytosolic levels rise [14]. PfCAX demonstrates Ca^2+^/H^+^ exchange activity, an ability to exchange a limited range of other divalent cations, and a high transport capacity but low affinity (K_m_ value of ∼2 mM) for Ca^2+^, when expressed in *Xenopus* oocytes [7]. *In vivo* studies characterising PfCAX are consistent with an atypical localisation to the inner mitochondrial membrane, and an atypical function, where the protein provides a pathway for removal of Ca^2+^ from this organelle back into the parasite cytosol.

The aim of this study was to determine the physiological importance of apicomplexan CAXs. In addition to developing a yeast heterologous expression system for the functional characterisation of apicomplexan CAXs, the genetically amenable *P. berghei* and *T. gondii* parasites were used with tagging and knock-out strategies to define expression and essentiality of their respective CAXs. The data demonstrate that under the control of their respective endogenous promoters only the expression of the tagged *P. berghei* CAX, PbCAX, could be established, and this, only in sexual stages of development (predominantly female specific). Furthermore, genetic disruption of *pbcax*, while having little or no effect on blood stage growth, was lethal during ookinete development. However, mutant parasites could be rescued by the removal of extracellular Ca^2+^.

## Results

### Sequence analysis of apicomplexan CAXs

**Figure 1:**
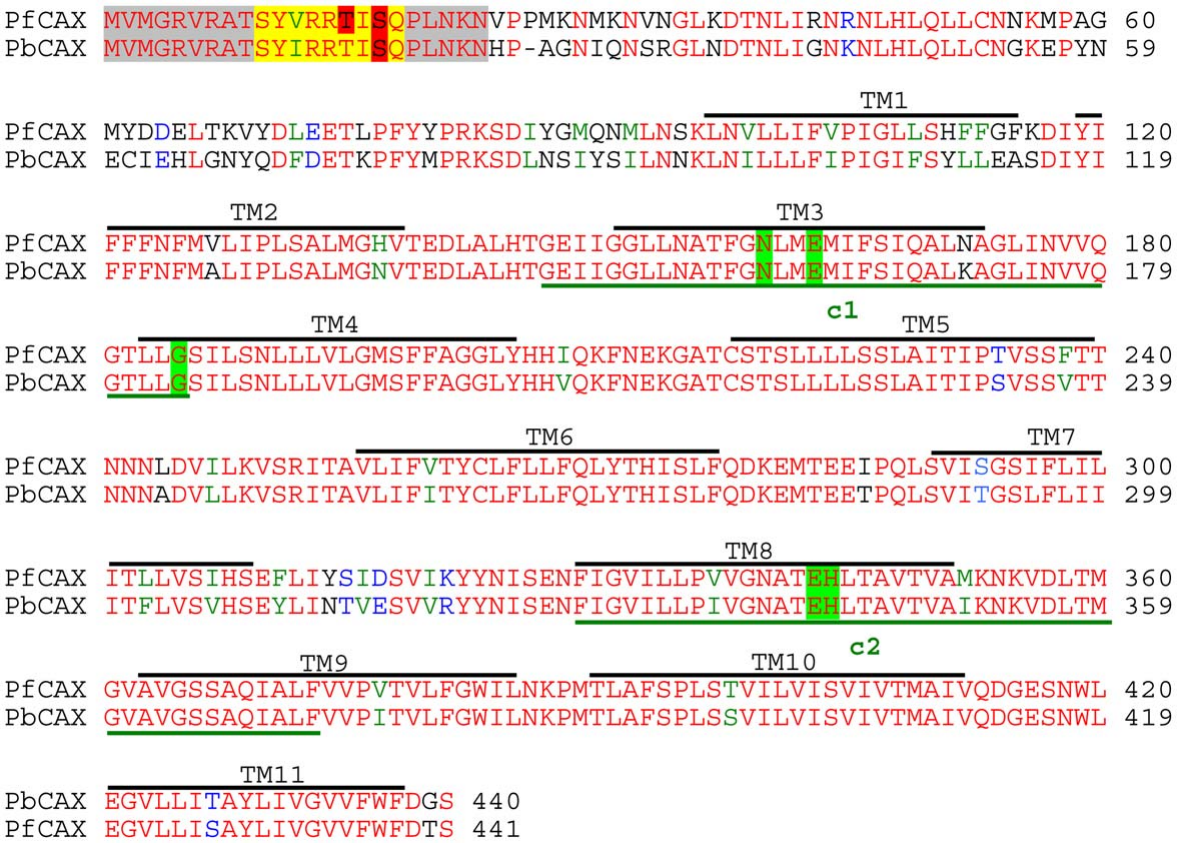
Sequence alignments. Amino acid sequence alignment of PfCAX with PbCAX. The Clustal W program was used to generate the alignment. The residues highlighted by a bold black line above correspond to transmembrane segment predictions determined with the TMHMM program (http://www.cbs.dtu.dk/services/TMHMM/). The residues highlighted by a bold green line below correspond to the conserved CAX regions, c-1 and c-2. Green shading denotes residues shown to be essential for Ca^2+^ transport in AtCAX1 and OsCAX1a [15], [16]. Yellow shading denotes the putative mitochondrial targeting motif [7]. Grey shading denotes cleaved sequences for mitochondrially imported proteins predicted by MitoProt II – v1.101 (http://ihg.gsf.de/ihg/mitoprot.html). Red shading denotes phospo-acceptor sites (GeneDB and [17]). CAX sequences are from (accession no.): Pf, *Plasmodium falciparum* (XP_966025.1) and Pb, *Plasmodium berghei* (XP_678577.1). *Red letters*, identical or conserved residues in all sequences; *green letters*, conserved substitutions; *blue letters*, semi-conserved substitutions.

The *P. falciparum*, *P. berghei* and *T. gondii* CAX genes, *pfcax* (PFF0170w), *pbcax* (PBANKA_010230) and *TgCAX* (TGME49_007910), have 1326, 1323 and 1506 base pair open reading frames, respectively, with only the latter having (12) introns. They are located on chromosomes 6, 1 and 1b in their respective genomes and encode polypeptides of 441, 440 and 501 amino acids, with estimated sizes of 48, 49 and 53 kDa, respectively (Figure 1 and S1). All the apicomplexan *cax* genes identified are single copy genes with no close paralogues. PfCAX has greater than 80%, approximately 50% and 39% amino acid sequence identity compared with other *Plasmodium* spp., *Coccidia* (*Toxoplasma*, *Cryptosporidium* and *Eimeria*) and *C. reinhardtii* CAX sequences, respectively. The phylogenetic relationship between the apicomplexan putative CAX transporters is shown in Figure S2, in which CrCAX1 has been added as the first functionally characterised Type 1-C CAX [10]. Interestingly, BLAST searches, using the PfCAX amino acid sequence, did not reveal *cax* genes in the genomes of the *Piroplasmida*, *Babesia bovis* and *bigemina* or *Theileria annulata*, even though they are closely related to *Plasmodium* parasites.

As with CrCAX1 and higher plant CAXs, the apicomplexan CAXs identified here are predicted to have 11 membrane spanning regions (with the TMHMM tool at www.cbs.dtu.dk/services/TMHMM/; Figure 1 and S1), although tagging experiments suggest that PfCAX spans the membrane 10 times only [7]. Apicomplexan CAXs also have long N-terminal sequences (ranging from 71 amino acids for *Cryptosporidium* ssp. CAXs to 126 amino acids for TgCAX). All contain conserved residues (Figure 1 and S1) that are essential for Ca^2+^ transport in *Arabidopsis thaliana* CAX1 and *Oryza sativa* CAX1a [15], [16] but not the non-consensus residues identified in the c-1 and c-2 repeat regions of CrCAX1 that may be involved in Na^+^ transport [10]. Also annotated on Figure 1 and S1 are the predicted mitochondrial targeting sequences and phospho-acceptor sites reported previously [7], [17] and on GeneDB (www.genedb.org) in the case of *P. berghei* ookinetes.

### Functional analysis of apicomplexan CAXs in yeast

To investigate whether apicomplexan CAXs can provide a tolerance mechanism against elevated Ca^2+^ concentrations and confirm that apicomplexan CAXs do function as Ca^2+^/H^+^ exchangers, a yeast heterologous expression approach was used. This was chosen over the *Xenopus* oocyte system used previously to study PfCAX [7], as our understanding of CAXs from many diverse organisms has been advanced more thoroughly through studies in yeast and for which there is i) a positive control, CrCAX1 [10], ii) optimised ways to detect function (see Materials and Methods), and iii) a context for interpretation of results [14]. CAXs were expressed in the *Saccharomyces cerevisiae* mutant K665, which lacks two vacuolar Ca^2+^ transport pathways; a Ca^2+^-ATPase (Pmc1) and a Ca^2+^/H^+^ exchanger (Vcx1). This yeast mutant is unable to sequester Ca^2+^ into the vacuole and is therefore hypersensitive to external Ca^2+^, which causes toxicity due to elevation of cytosolic Ca^2+^ concentrations [11].

A codon optimized *pfcax* cDNA (Figure S3) was expressed in K665 yeast and the ability of PfCAX to provide expressing yeast tolerance to high Ca^2+^ stress was assessed. Ca^2+^ tolerance by PfCAX was compared alongside the previously characterised Ca^2+^/H^+^ exchanger from *Chlamydomonas* [10]. The 2009 study demonstrated that the *Chlamydomonas* exchanger was more efficient at transporting Ca^2+^ when expressed in yeast if the N-terminal tail is truncated, removing a putative regulatory domain. An N-terminal truncated variant of *pfcax* (*spfcax*), in which translation was initiated from AUG at nucleotide position 181 (encoding Met-61), was therefore generated for comparison with PfCAX and sCrCAX1.

**Figure 2:**
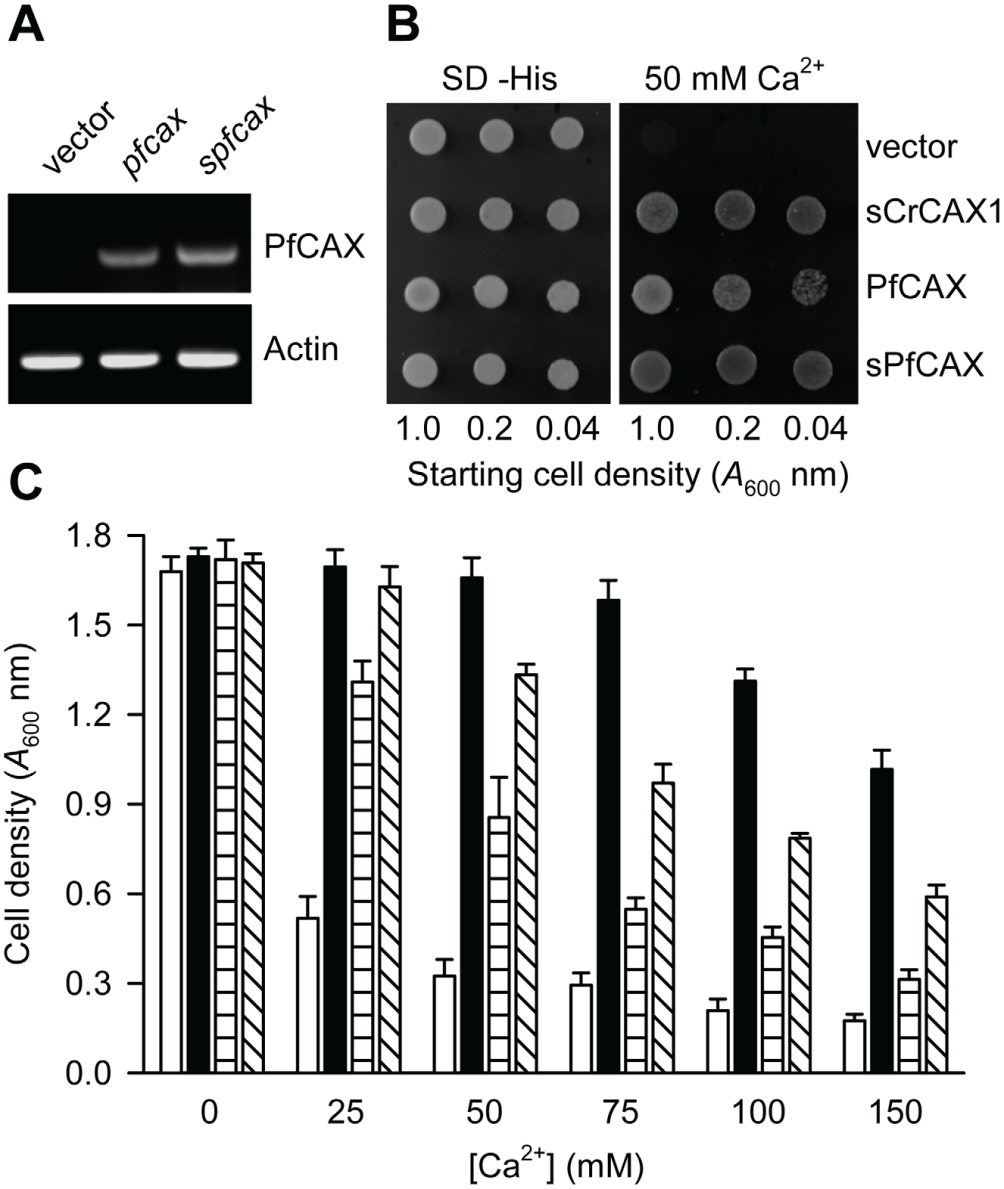
Ca^2+^ tolerance of yeast mediated by PfCAX. (A) RT-PCR analysis of *pfcax* and *spfcax* expression in yeast compared with yeast transformed with the empty vector control. (B) Saturated liquid cultures of K665 (*pmc1 vcx1*) yeast transformed with *pfcax* in piHGpd, N-terminally truncated s*pfcax* in piHGpd, *scrcax1* in piHGpd and empty vector alone were serially diluted to the cell densities as indicated, then spotted onto selection medium lacking histidine (SD –His) and YPD medium containing 50 mM CaCl_2_. Yeast growth at 30°C is shown after 4 days. A representative experiment is shown. (C) K665 yeast transformed with the various plasmids described in (B) were diluted to a cell density of 0.5 *A*_600_ nm and inoculated into YPD medium containing concentrations of CaCl_2_ from 25 to 150 mM. Yeast cell density was determined by absorbance measurements at 600 nm following growth shaking at 30°C for 16 h. Bars represent the mean ± SEM of 4 independent experiments (each with 8–12 replicates). Vector only, open bars; sCrCAX, closed bars; PfCAX, horizontally lined bars; sPfCAX, diagonally lined bars.

Expression of *pfcax* and *spfcax* in yeast was detectable by RT-PCR (Figure 2A). Comparison of PfCAX, sPfCAX, sCrCAX1 and empty vector expressed in yeast grown on YPD (yeast-peptone-dextrose) media supplemented with 50 mM CaCl_2_ (Figure 2B) found that sPfCAX could suppress the Ca^2+^ hypersensitivity of the yeast mutant as efficiently as sCrCAX1, while full-length PfCAX-expressing yeast growth was slightly reduced (as determined by the non-uniform growth of yeast expressing PfCAX at a starting cell density of 0.04 absorbance units at A_600_ nm). The Ca^2+^ tolerance of yeast expressing PfCAX and sPfCAX was demonstrated further by cell growth in liquid YPD media supplemented with various concentrations of CaCl_2_ (Figure 2C). sPfCAX provided K665 yeast with tolerance to high concentrations of CaCl_2_. sPfCAX-expressing yeast growth was significantly greater than PfCAX-expressing yeast at each CaCl_2_ concentration (p<0.02 unpaired, two-tailed Student's *t*-test; n = 4), while the cell growth of PfCAX-expressing yeast was significantly greater than that of the empty vector control yeast at all CaCl_2_ concentrations, including at 150 mM CaCl_2_ (p<0.02, unpaired, two-tailed Student's *t*-test; n = 4). A truncated version of *TgCAX* (*sTgCAX*), in which translation was initiated by introducing an AUG prior to nucleotide position 295 (encoding Ala-99), was also generated and expressed in K665 yeast. The transformation was assessed to have worked by PCR (Figure S4A) and, as with PfCAX and sPfCAX, sTgCAX suppressed the Ca^2+^ hypersensitivity of the yeast (Figure S4B). These data demonstrate that apicomplexan CAXs and truncated variants are functional in yeast, and in this heterologous system, can function in providing Ca^2+^ tolerance.

**Figure 3:**
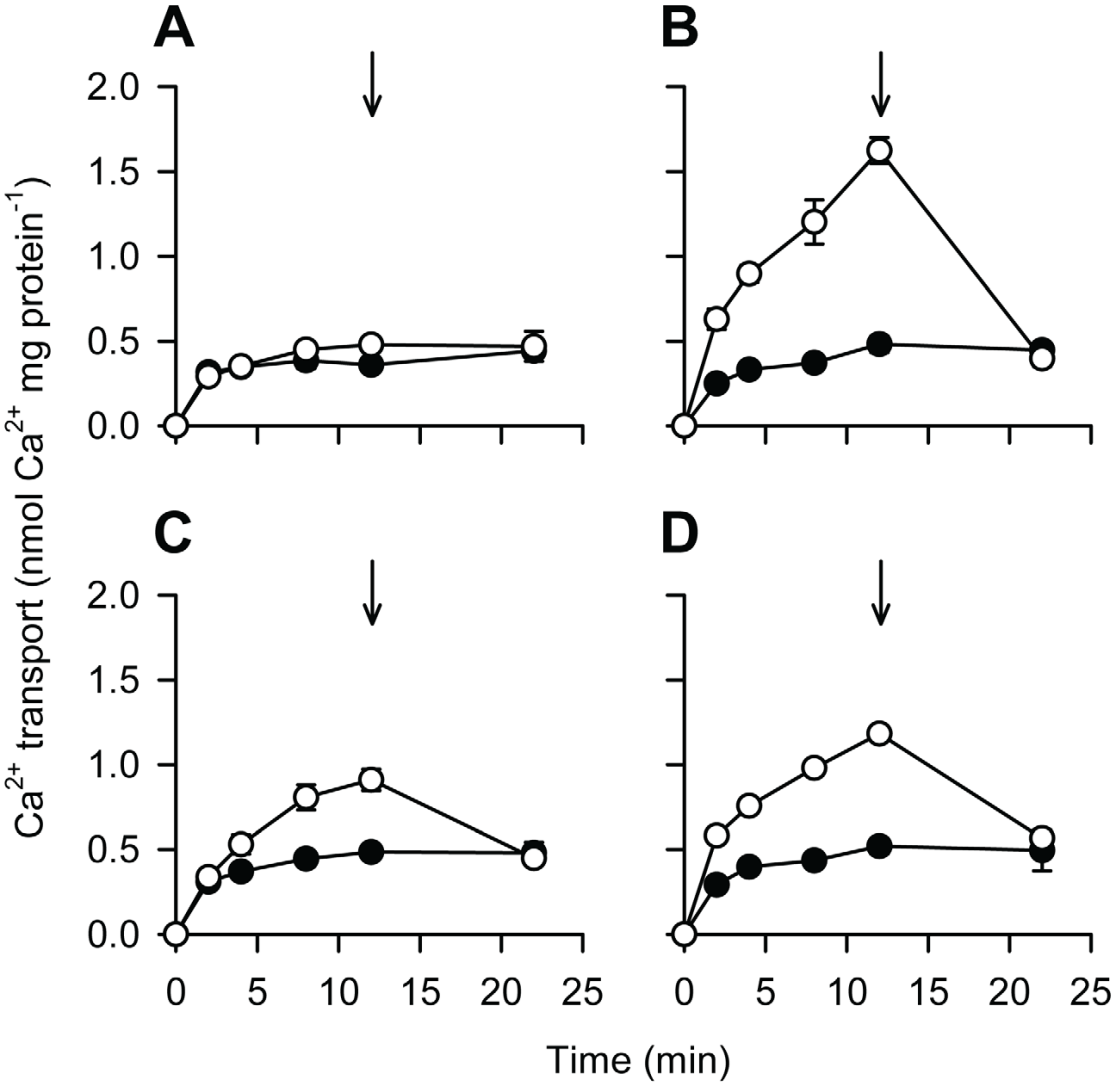
Ca^2+^/H^+^ exchange activity of PfCAX. ΔpH-dependent uptake of 10 µM ^45^Ca^2+^ into vacuolar-enriched membrane vesicles isolated from K665 (*pmc1 vcx1*) yeast transformed with empty vector (piHGpd) (A), *scrcax1* (B), *pfcax* (C), and *spfcax* (D), measured over a 22 min time course. Transport measurements were determined in the presence of 0.1 mM NaN_3_, 10 mM KCl, 1 mM ATP, 1 mM MgSO_4_ and 0.2 mM orthovanadate (a Ca^2+^-ATPase inhibitor). ^45^Ca^2+^ uptake in the absence (open symbols) or presence (closed symbols) of 5 µM of the protonophore CCCP is shown. The Ca^2+^ ionophore A23187 was added at 12 min at a concentration of 5 µM to dissipate any vesicle-loaded ^45^Ca^2+^, as indicated by arrows. Points represent the mean ± SEM of 4–5 independent experiments (where not shown errors lie within the symbols).

To confirm that growth of PfCAX-expressing yeast on Ca^2+^-containing media was due to enhanced vacuolar Ca^2+^/H^+^ exchange activity, ΔpH-dependent ^45^Ca^2+^ uptake in the presence of the Ca^2+^-ATPase inhibitor vanadate was examined in vacuolar membrane vesicles isolated from K665 yeast expressing each of the *cax* plasmids. ΔpH across the vacuolar membrane vesicles was established by activation of endogenous H^+^-ATPase activity by the addition of Mg^2+^-ATP. Significant Ca^2+^/H^+^ exchange activity, which could be inhibited by the protonophore carbonyl cyanide 3-chlorophenyl hydrazone, CCCP, was measured for each CAX variant (Figure 3). However, sPfCAX activity was reduced compared with that of sCrCAX1 (by 42% at the 12 min time point; 0.662±0.032 versus 1.142±0.076 nmol mg protein^-1^, after subtraction of basal accumulation; p = 0.0004, unpaired, two-tailed Student's *t*-test; n = 5). Ca^2+^/H^+^ exchange activity mediated by full-length PfCAX was significantly reduced compared with that of sPfCAX (36% reduced Ca^2+^ uptake at 12 min time point; 0.425±0.063 versus 0.662±0.032 nmol mg protein^−1^, after subtraction of basal accumulation; p = 0.01, unpaired, two-tailed Student's *t*-test; n = 5) but was detectable over basal (empty vector) levels (p = 0.0007, unpaired, two-tailed Student's *t*-test; n≥4).

### Expression profile of PbCAX-GFP during the *P. berghei* life cycle

Previously, transient expression of a green fluorescence protein (GFP)-tagged version of *pfcax*, using a strong but non-authentic/non-sequential promoter, was used to localise PfCAX to the parasite mitochondrion [7]. To enable expression and localisation of a *Plasmodium* CAX to be determined over the parasite's full life cycle, the rodent parasite, *P. berghei*, was used. A GFP-tagged *P. berghei* parasite line was generated by endogenous C-terminus fusion of the *gfp* sequence onto *pbcax*, leaving control of gene expression under the endogenous promoter. This was achieved with a single crossing over homologous integration approach (Figure S5A). PCR on genomic DNA obtained from a tagged clone was positive for integration of the tagging construct (using primers INT N43tag and ol492; Table S1, Figure S5B and Protocol S1) and pulse field gel electrophoresis demonstrated that the integration occurred, as expected, on chromosome 1 (Figure S5C and Protocol S1).

Expression of PbCAX-GFP in activated gametocytes was confirmed by Western blotting in the particulate fraction (contain membranes), using anti-GFP polyclonal antibodies (Figure S5D and Protocol S1). A protein band of ∼77 kDa was identified in samples of PbCAX-GFP expressing parasites, which corresponds to the predicted mass of the PbCAX-GFP fusion protein. No smaller bands could be identified, suggesting that the tagged protein remains intact *in situ*. As a positive control for blotting and antibody staining, a *P. berghei* line that constitutively expresses GFP in the cytosol throughout the life cycle was used [18]. An appropriate protein band of approximately 29 kDa (the predicted size of GFP) was observed in the supernatant fraction derived from these parasites (note that a similarly sized band was also present in the particulate fraction, which may represent contamination from the supernatant).

Using epi-fluorescence microscopy in an initial screen of live parasite stages, only very low level, diffuse fluorescence signal was observed, predominantly, in asexual blood stages of *pbcax-gfp* transgenic parasites, with stronger parasite-associated GFP signal observed only on rare occasions (data not shown). Stronger GFP signal was observed in female but not male gametocytes and the same was true for gametes (Figure S6A). Thus, the asexual parasites with stronger GFP signal may have been immature female gametocyte stages. Good GFP signal was also observed in zygotes, ookinetes and oocysts.

**Figure 4:**
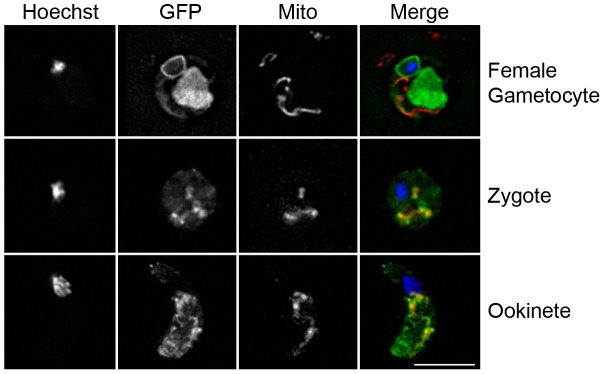
PbCAX-GFP localisation. High resolution deconvolution microscopy images of a live female gametocyte shortly after activation and a female gamete/zygote and an ookinete 24 h post activation expressing PbCAX-GFP and co-stained with MitoTracker Red CMXRos (10 ng/ml) and Hoechst 33342. Scale bar: 5 µm.

To improve resolution of live parasite fluorescence images, deconvolution microscopy was used. In activated gametocytes, GFP signal was confined to membranous regions surrounding the parasite nucleus and a mass to the side (possibly the endoplasmic reticulum). Little signal colocalised with MitoTracker, used as a marker for parasite mitochondrion (Figure 4). Non-converting female gametes/zygotes and ookinetes 24 h after activation had more dispersed GFP signal and only very partial colocalisation with the mitochondrion could be observed (Figure 4). Additional images of the latter stages co-stained with a parasite surface marker rather than MitoTracker can be seen in Figure S6B.

### PbCAX is not essential for the erythrocytic stages of *P. berghei*

To determine the physiological importance of Ca^2+^/H^+^ exchange activity during the erythrocytic stages of *P. berghei*, a direct *pbcax* knock-out strain was generated by double crossing over homologous recombination (Figure S7A). After transfection of *P. berghei* ANKA and a second line that constitutively expresses cytosolic GFP [18] with a PbCAX knock-out vector, parasites containing the knock-out construct were selected by pyrimethamine drug pressure. PCR on genomic DNA obtained from two independent clones derived from each of the parasite lines (*cl9* and *cl5 gfp*) were both positive for integration of the knock-out construct (using primers INT N43 and ol248) and disruption (using primers N43 KO1 and N43 KO2) of *pbcax* (Table S1, Figure S7B and Protocol S1). Further confirmation that the gene deletion was successful came from Southern hybridisation of digested genomic DNA (Figure S7C and Protocol S1) and pulse field gel electrophoresis, using a probe that recognises the resistance marker (Figure S7D and Protocol S1). The latter demonstrates that the integration occurred on the correct chromosome (i.e. chromosome 1). The ability to derive these clones and their apparently normal *in vivo* asexual blood-stage growth and gametocyte production (not tested quantitatively) suggest that PbCAX function is not essential for blood stages of the *P. berghei* life cycle.

### PbCAX is essential for sexual stage (ookinete) development *in vitro*

**Figure 5:**
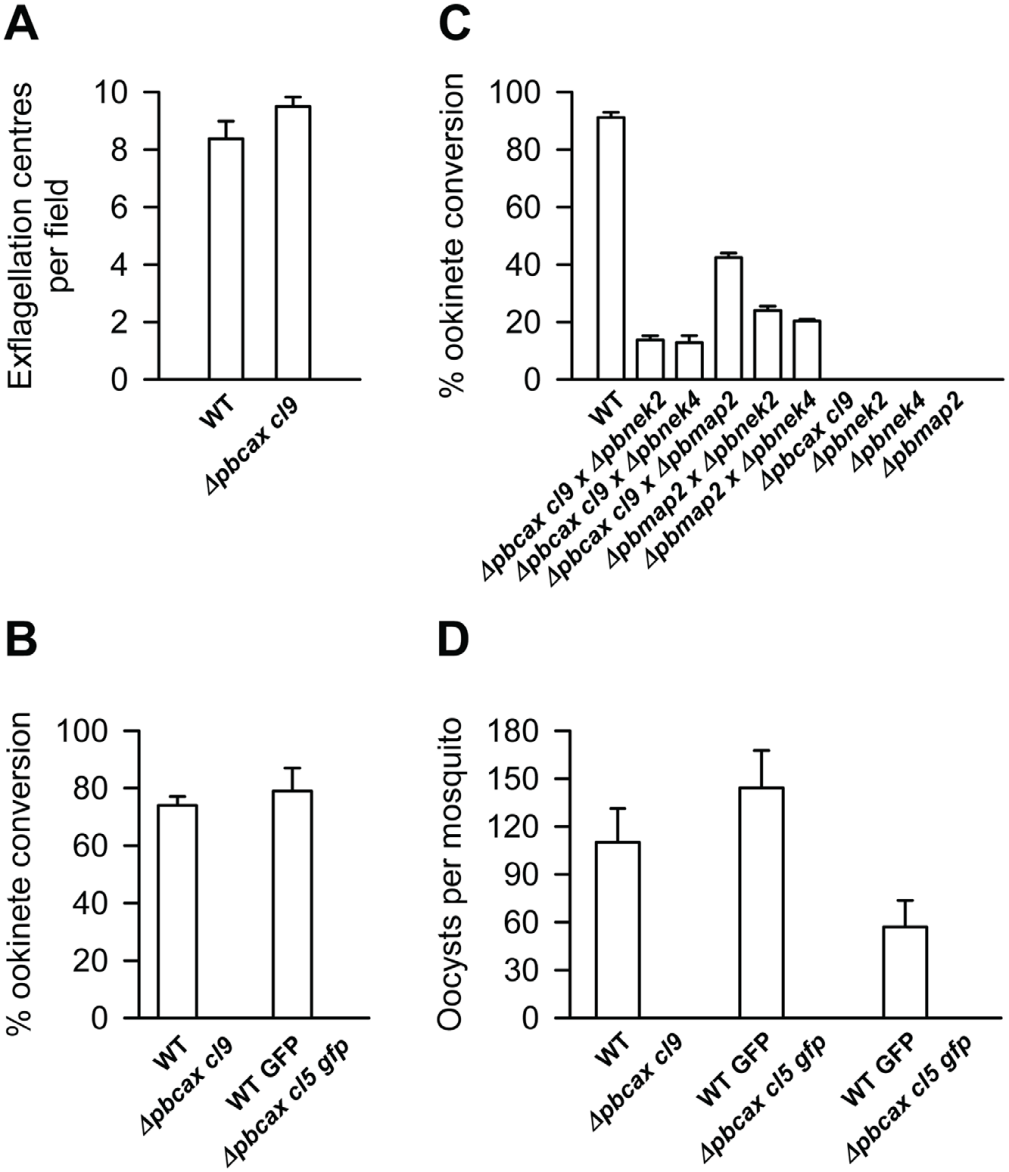
*Δpbcax* parasite phenotype. (A) Bar graph illustrating exflagellation in wild-type (WT) and *Δpbcax cl9* parasites. Exflagellation is presented as the numbers of exflagellation centres in 8 fields of view (magnification, ×40). Bars represent the mean ± SEM of 3 independent experiments. (B) Bar graph illustrating ookinete conversion in wild-type (WT or WT GFP) and *Δpbcax cl9 and cl5 gfp* parasites. The conversion rate is the percentage of P28-positive parasites that had successfully differentiated into elongated ‘banana-shaped’ ookinetes. Bars represent the mean ± SEM of 3–4 independent experiments except for WT GFP, where the bar represents the mean ± range (n = 2). (C) Ookinete conversion after crossing *Δpbcax cl9* parasites with female-defective *nek2* and *nek4* mutants (*Δpbnek2/4*) and a male-defective *map2* mutant (*Δpbmap2*). Wild-type parasites (WT) were used as a control. The conversion rate is the percentage of P28-positive parasites that had successfully differentiated into elongated ‘banana-shaped’ ookinetes. Bars represent the mean ± SEM of 3 independent experiments. (D) Bar graph illustrating the mean ± SEM numbers of oocysts per midgut (20 analysed) of wild-type (WT or WT GFP) and either *Δpbcax cl9* or *cl5 gfp* infected mosquitoes in three independent experiments.

Next, the *in vitro* sexual development of mutant parasite clones was studied [19]. Firstly, the ability of male gametocytes to undergo exflagellation was tested by adding gametocyte containing blood to “activation” ookinete medium (see Methods and Materials) and counting exflagellation centres. Figure 5A presents data, using *cl9 Δpbcax* parasites, in which no statistical difference was determined between the number of exflagellation centres produced by wild-type parasites compared with the mutant line (8.4±0.6 versus 9.5±0.3; mean ± SEM; n = 3; p = 0.2, unpaired, two-tailed Student's *t*-test). Apparently normal exflagellation was also observed for *cl5 gfp Δpbcax* parasites (not tested quantitatively).

Secondly, ookinete conversion was determined by culturing parasites for 24 h in ookinete medium and measuring the number of the motile ookinete forms in relation to unconverted “round” forms (female gametes/zygotes) at the end of this period, using fluorescently-labelled P28 antibody to aid identification (note that P28 is a surface expressed antigen found on activated female gametes, zygotes and ookinetes). Figure 5B presents the data from three experiments using *cl9 Δpbcax* parasites in which no ookinete conversion was observed, while wild-type control parasites converted to ookinetes on average by 74±3% (mean ± SEM; n = 3). No ookinete conversion was also observed for *cl5 gfp Δpbcax* parasites (n = 4), while in two matched experiments, using wild-type control parasites that constitutively express GFP, estimated conversion rates were 87 and 71%. Note that very occasional ookinete-like forms were observed in these *in vitro* cultures.

**Figure 6:**
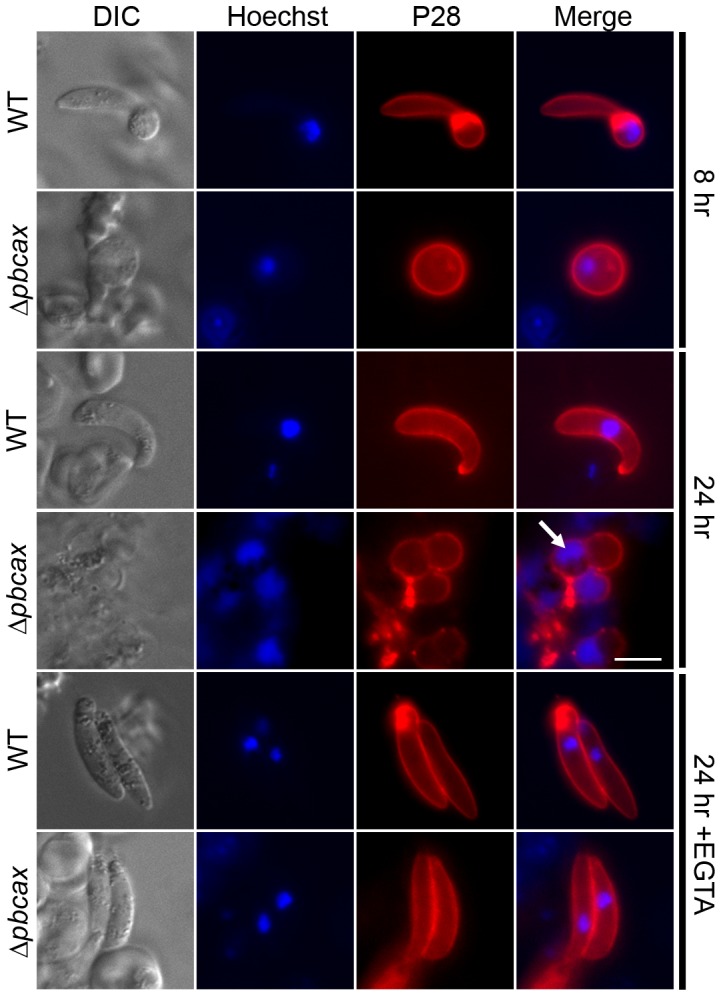
*Δpbcax* parasite ookinete conversion images. Immunofluorescence images of *in vitro* wild-type (WT) and *Δpbcax cl9 P. berghei* ookinete cultures, 8 and 24 h after gametocyte activation and in the presence of EGTA (10 mM; added directly prior to gametocyte activation). Parasites are immunostained for the female gamete/zygote/ookinete marker P28 (red) and co-stained with the nuclear marker Hoechst 33342 (blue). Development of elongated ookinetes was completely ablated in the *Δpbcax cl9* line, a phenotype which could be reversed by the removal of extracellular Ca^2+^ using EGTA. The arrow indicates the diffuse DNA staining observed in *Δpbcax cl9* parasites 24 h after gametocyte activation. Scale bar: 5 µm.

Figure 6 shows examples of the typical phenotype that was observed for the mutant parasites. At 8 h post-activation, wild-type parasites were predominantly “retort” forms (a round parasite that contains the nucleus, with an apical protrusion), whereas *Δpbcax* parasites remained round. At 24 h post-activation, wild-type parasites were predominantly fully converted into ookinetes (elongated forms with the nucleus in the centre). At this time, *Δpbcax* parasites were still round in form, fewer in number and were often smaller in size and had degenerated membranes (as judged from discontinuous P28 staining). Furthermore, they often had diffuse nuclei, suggestive of possible necrosis or late stage apoptosis. In a single semi-quantitative experiment with three repeats, the number of “round” form PbCAX knock-out parasites per field of view was counted in ookinete development cultures at 2, 6 and 24 h after activation (Figure S8). An approximate 70% reduction in the number of parasites present after 24 h was observed, suggesting that the parasites are degrading rather than arrested or slow growing.

### *Δpbcax* phenotype is carried preferentially by activated female gametes/zygotes

Cross-fertilization experiments were performed in an effort to establish if PbCAX activity is an important male and/or female trait (Figure 5C). This was performed by co-culturing *Δpbcax cl9* gametocytes with gametocytes of either the female-defective *nek-2* or *nek-4* null mutants [20], [21] or a male-defective *map-2* null mutant [22] and determining ookinete conversion. While none of the mutants alone underwent ookinete conversion, crossing *Δpbcax* with *Δnek2 or Δnek4* resulted in recovered ookinete conversion of 14±1 and 13±2%, respectively (mean ± SEM; n = 3). This is due to the fertilisation of female *Δpbcax cl9* parasites by functional *Δnek2* or *Δnek4* male gametes. However, crossing *Δpbcax cl9* with *Δmap2* resulted in recovered ookinete conversion of 42±2% (mean ± SEM; n = 3). This is due to the fertilisation of functional *Δmap2* female gametes by male *Δpbcax* parasites. These data suggest that PbCAX activity, while not specific, is predominantly important to female gametes.

### PbCAX is essential for parasite transmission to the mosquito *in vivo*

Parasite development was assessed *in vivo* by feeding *Anopheles stephensi* mosquitoes on mice infected with *Δpbcax cl9* or *cl5 gfp* parasite mutants and measuring oocyst formation in the mosquito midgut 14 days later. Figure 5D shows three independent experiments, the first using *Δpbcax cl9* and the second two using *Δpbcax cl5 gfp* parasites (with 20 mosquito midguts analysed per assay). It demonstrates that while mosquito infection with wild-type parasites produced mean numbers of oocysts per mosquito midgut of 110, 144 and 57, respectively, and infection prevalences (number of mosquitoes with oocysts) of 80, 90 and 65%, infection with parasites lacking PbCAX produced none. At day 21 post-infection, the infected mosquitoes were used to re-infect mice but no mutant asexual blood-stage parasites could be identified after 15 days, while wild-type asexual blood-stage parasites were observed by day 5 on average.

### *Δpbcax* phenotype can be rescued by removal of extracellular Ca^2+^

**Figure 7:**
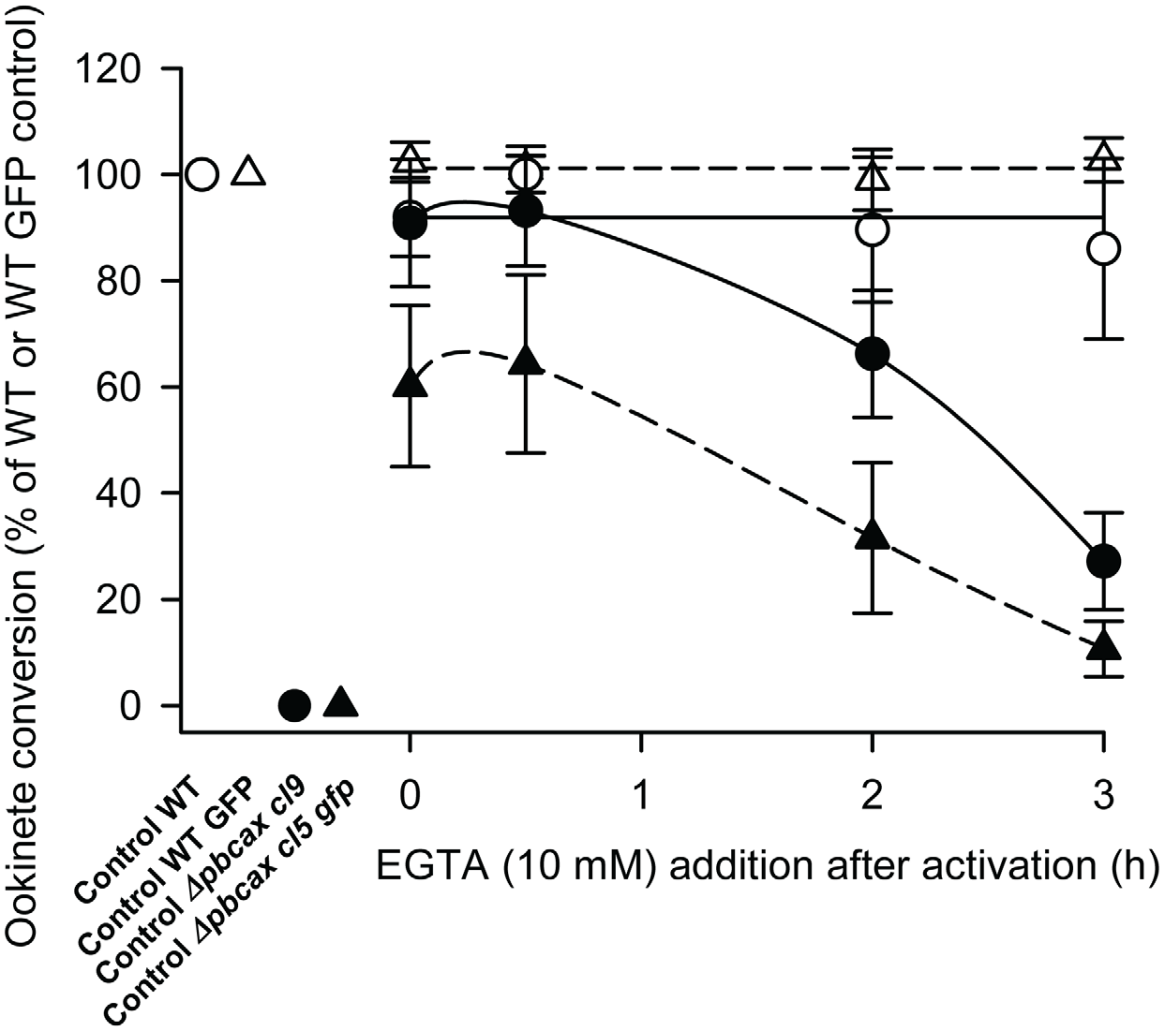
*Δpbcax* parasite rescue with EGTA. Line graph illustrating ookinete conversion, measured at 24 h post-gametocyte activation, in wild-type (WT, open circles: WT GFP, open triangles) and *Δpbcax cl9* (closed circles) and *cl5 gfp* (closed triangles) parasites in the presence of 10 mM EGTA added at 0, 0.5, 2 and 3 h post-gametocyte activation. The conversion rate is the percentage of P28-positive parasites that had successfully differentiated into elongated ‘banana-shaped’ ookinetes. Data are expressed as the percentage of wild-type controls. Points represent the mean ± SEM (n = 3) except for WT, where the points represent the mean ± range (n = 2).

Given that transmission stages of parasite development are extracellular (the *in vitro* culture medium used here contained 0.42 mM Ca^2+^) and that CAX activity often provides a mechanism to enable organisms to survive in the presence of extracellular Ca^2+^ [14], we tested the ability of the Ca^2+^ chelator EGTA (ethylene glycol tetraacetic acid) to restore ookinete conversion of both *Δpbcax cl9* and *cl5 gfp* parasites (Figure 7). EGTA (10 mM) had no significant effect on the normal ookinete conversion efficiency of wild-type parasites, which constitutively expressed GFP (control parasites used for comparison with *Δpbcax cl5 gfp*; open triangles in Figure 7), measured at 24 h post-activation when added immediately prior to gametogenesis (t = 0 h), 30 min post-activation (at which point exflagellation has occurred), 2 h post-activation (at which point zygote formation will be complete, predominantly) or 3 h post-activation (p>0.05, ANOVA with Dunnett's post test; n = 3). Apparently similar results were found for the development of non-GFP expressing wild-type parasites (control parasites used for comparison with *Δpbcax cl9*; open circles in Figure 7) grown in the presence of EGTA, although this could not be tested statistically as only 2 control experiments were performed. The effect of EGTA on *Δpbcax cl9* and *cl5 gfp* parasites was to restore ookinete conversion, when added 3 h post-activation or prior to this point, although the ability to restore ookinete conversion reduced the longer after activation EGTA was added. In the case of all *Δpbcax cl9* experiments and one of the three using *Δpbcax cl5 gfp* parasites, addition of EGTA at 0 and 0.5 h led seemingly to complete restoration of ookinete conversion. Images of restored *Δpbcax cl9* ookinetes in the presence of EGTA added at t = 0 h can be seen in the lower panels of Figure 6. Addition of EGTA at 3 h post-activation still enabled ookinete conversion of *Δpbcax cl9* and *cl5 gfp* parasites but at significantly lower levels than the GFP-expressing wild-type control (p<0.05, ANOVA with Dunnett's post test; n = 3). In case the rescuing effect of EGTA was osmotic rather than due to its Ca^2+^ chelation properties, additional controls were performed in the presence of 20 mM NaCl added to the culture medium immediately prior to gametogenesis. This had no effect on the ability of wild-type parasites to develop into ookinetes (p = 0.6, unpaired, two-tailed Student's *t*-test; n = 3) or the inability of mutant parasite to develop (data not shown).

### Localisation of TgCAX-Ty in *T. gondii*

**Figure 8:**
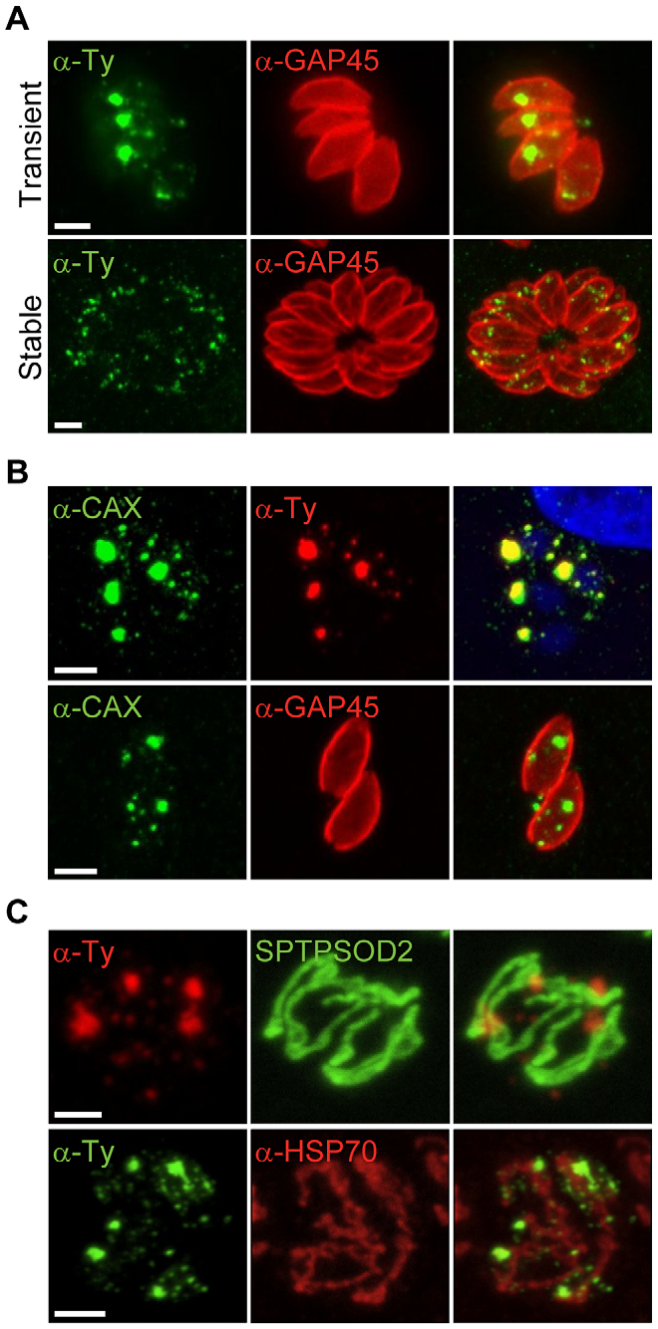
TgCAX localisation. (A) Immunofluorescence assay of intracellular *Toxoplasma* tachyzoites transiently transfected with *TgCAX-Ty* expressed under the control of the tubulin promotor (upper panels) and when expressed stably (lower panels). Parasites are immunostained with the surface marker GAP45 (red). (B) TgCAX transiently transfected tachyzoites detected by anti-TgCAX antibodies and co-stained with anti-Ty antibodies and the nuclear marker DAPI (upper panels) or with GAP45 antibodies (lower panels). (C) TgCAX transiently expressed in tachyzoites co-localised with 2 mitochondrial markers, anti-HSP70 antibodies [26] (upper panels) or transiently co-transfected SPTPSOD2GFP (SP: signal peptide, TP: transit peptide, [27]) (lower panels). Scale bars: 2 µm.

In a highly complementary approach to allow comparative tagging and knock-out studies with *P. berghei*, additional experiments were performed using the genetically amenable apicomplexan, *T. gondii*, at the tachyzoite stage. When TgCAX was expressed transiently under the control of the tubulin promoter, as a second copy detectable by a C-terminal Ty-tag (Figure S9A), the protein was found predominantly in a large vesicular-like compartment located in the apical end of the intracellular parasite, as well as in much smaller vesicle-like structures dispersed throughout the parasite cytosol (Figure 8A). This compartment is reminiscent of the plant-like vacuole (PLV) described recently in extracellular tachyzoites [23]. However, there was no co-localization with antibodies directed against a marker for the PLV and acidocalcisomes, the vacuolar proton pyrophosphatase, VP1 (Figure S9B). When TgCAX-Ty was stably expressed in a pool of intracellular parasites, only the dispersed signal was observed (Figure 8A), similar but seemingly not identical to that reported for VP1 [23].

Antibodies raised against an N-terminal region of the protein colocalised with the transiently expressed TgCAX-Ty (Figure 8B). However, in intracellular wild-type parasites no signal was detected by immunofluorescence, using the N-terminal directed antibodies. Although proteomic data from tachyzoite stage preparations include TgCAX (*e.g.* [17]), it may not be expressed at a detectable level by IFA at this stage. To test this hypothesis, the endogenous *TgCAX* was tagged with a 3Ty-tag (Figure S9C). This was introduced at the C-terminus of *TgCAX* by a knock-in strategy, using the ΔKU80 strain of *T. gondii* [24], [25]. In this strain, the non-homologous end-joining DNA repair pathway is defective and therefore integration essentially occurs only by homologous recombination at the targeted locus. The integration was confirmed by RT-PCR (Figure S9D) but no signal was detected by immunofluorescence (data not shown), supporting the hypothesis that the protein is expressed at a very low level during this stage of the parasite's life cycle. Furthermore, transiently expressed Ty-tagged TgCAX shows no colocalization with HSP70 or with co-transfected SOD2-GFP (Figure 8C) that label specifically the mitochondrion of *T. gondii* [26], [27].

### TgCAX is not essential for the tachyzoite stage of *T. gondii*

**Figure 9:**
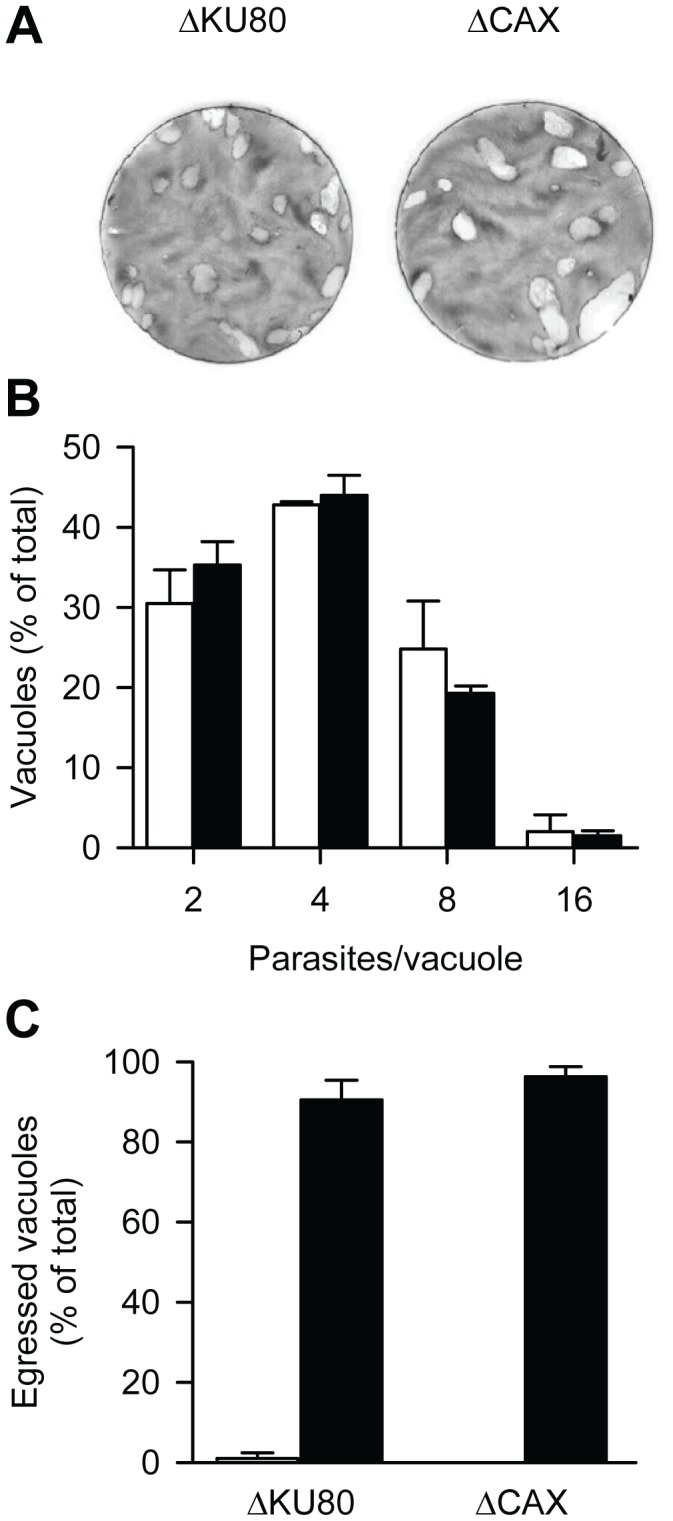
Essentiality of TgCAX in *T. gondii* tachyzoites. (A) Plaque assays were performed by incubating host cells with ΔKU80 and ΔCAX parasite strains for 7 days. (B) For the intracellular growth assay, ΔKU80 (open bars) and ΔCAX (closed bars) parasites were grown in host cells for 24 h and then the number of parasites per vacuole was determined. Bars represent the mean ± SEM of 3 independent experiments (each with 2 replicates). (C) For the ionophore-induced egress assay, parasites were cultured for 30 h before treatment with DMSO (open bars) or Ca^2+^-ionophore A23187 (3 µM; closed bars) for 5 min. The results are expressed as a percentage of ruptured vacuoles. Bars represent the mean ± SEM of at least 2 independent experiments.

To determine the physiological importance of Ca^2+^/H^+^ exchange activity during the tachyzoite stage of *T. gondii*, a similar approach to that used for the study *P. berghei* was taken (Figure S9E and S9F). Plaque assay experiments were performed and revealed no obvious defect in the lytic cycle of the *ΔTgCAX* strain compared with wild-type parasites after 7 days (Figure 9A). To confirm this observation, intracellular growth and egress were assessed specifically. In the case of intracellular growth, the distributions of parasites per vacuole were essentially identical for both the *ΔTgCAX* and wild-type strains (Figure 9B) and parasite egress induced by the Ca^2+^ ionophore A23187 was also similar between strains (Figure 9C). These data suggest that TgCAX is dispensable during the tachyzoite stage.

## Discussion

Given the importance of Ca^2+^ in numerous essential cellular roles during the life cycles of apicomplexan parasites [1]–[3], understanding Ca^2+^ homeostasis could lead to the development of novel parasiticidal strategies. Here, the physiological importance of two apicomplexan CAXs has been examined.

### Functional characterisation of apicomplexan CAXs

Nearly all *cax* genes characterised to date from bacteria, fungi and plants encode H^+^-coupled Ca^2+^ transporters [11], [12], [28]. While some CAX transporters are highly specific for Ca^2+^, such as yeast Vcx1, others have a broader ion specificity and can mediate the transport of different divalent cations [13], [15] or can transport both divalent (*e.g.* Ca^2+^) and monovalent (*e.g.* Na^+^) cations, in the case of CrCAX1 [10].

Previous characterisation of PfCAX by expression in *Xenopus* oocytes reported Ca^2+^/H^+^ exchange and, using competition assays, data implicating transport of other divalent cations, including Mg^2+^ and transition metal ions [7]. Here, we developed an alternative approach to characterise PfCAX and TgCAX, taking advantage of the yeast expression system that is most commonly used to study CAXs (*e.g.* [10]). Expression of PfCAX and (N-terminally truncated) sPfCAX and sTgCAX in a yeast strain lacking vacuolar Ca^2+^ transporters increased tolerance of yeast to extracellular Ca^2+^. Transport measurements in yeast vacuolar membrane preparations confirmed that PfCAX is a Ca^2+^/H^+^ exchanger and is localised to the tonoplast when expressed in yeast. In a previous study [7] there was no physiological interaction of PfCAX with Na^+^, as would be predicted because sequences that maybe associated with Na^+^ transport [10] are lacking in the PfCAX sequence unlike CrCAX1. In agreement with previous work demonstrating that the N-terminal region of plant CAXs is autoinhibitory [29], N-terminal truncation of PfCAX improved Ca^2+^ tolerance and Ca^2+^ transport rates. Truncation of the protein may also improve delivery to the vacuole and provide an alternative explanation for improved functionality.

While this paper was under revision, a report describing PfCAX/CHA expression in yeast lacking the endogenous CAX gene (*vcx1*) and containing a calcium biosensor (apoaequorin) was published [30]. Although no new functional data were reported and only indirect transport measurements were possible, unlike the data presented here (see Figure 3), the data produced, using this system, are consistent with PfCAX functioning as a Ca^2+^/H^+^ exchanger and lend support to the present findings.

### Localisation of apicomplexan CAXs

While functional characterisation of proteins, whether *in situ* or in heterologous expression systems, provides an understanding of what a protein does, localisation of proteins *in situ* adds physiological context. Most plant and fungal CAXs localise to the tonoplast and provide efflux pathways to remove Ca^2+^ from the cytosol. In the case of PfCAX, *Xenopus* oocyte [7] and yeast (this study and [30]) heterologous expression data suggest plasma membrane and tonoplast localisation, respectively. However, tagging studies indicate that PfCAX is located in the parasite's mitochondrial inner membrane [7], a novel CAX localisation. Given these complexities, additional expression and localisation studies were performed in *T. gondii* and *P. berghei* parasites.

While proteomic analysis suggests that TgCAX is present in intracellular *T. gondii* tachyzoites (*e.g.* [17]), tagging of the gene under control of the endogenous promoter did not allow localisation, as expression may be limited at this stage. However, use of a strong promoter by transient transfection enabled localisation of TgCAX, mainly to a large vesicular-like region, with similar size, shape and location to the PLV, as described previously [23]. Considering that the staining pattern covered the entire region of the vesicle-like structure, rather than just the edge (as might be expected for integral proteins such as CAXs), the lack of co-localisation with VP1 (a marker of both the PLV and acidocalcisomes) and the loss of this staining pattern over time, it may be that these staining results are an artifact of overexpression. There was also no evidence for mitochondrial localisation of TgCAX (see Figure 8), as there is for PfCAX [7]. Stably transfected parasites contained punctate staining for tagged TgCAX. The punctate staining pattern with TgCAX-Ty, although consistent with what is observed for acidocalcisomes, did not co-localise with VP1. This suggests that TgCAX-Ty may localise to a separate novel vacuolar compartment such as one containing cathepsin L [31] and will benefit from further studies.

Similar to TgCAX, there is proteomic evidence that PfCAX is expressed during the asexual blood stage (*e.g.* [17]). Here, the expression of PbCAX-GFP was barely detectable in asexual blood stages. This is consistent with the low level of expression of TgCAX and the apparently inessential nature of PbCAX and TgCAX at similar life cycle stages. PbCAX-GFP expression was much greater in gametocytes (predominantly females) and this expression profile was maintained throughout the sexual stages. Localisation altered during sexual development. In activated female gametes, the data are consistent with a build-up of PbCAX-GFP within a large membranous network that is most likely the endoplasmic reticulum (although other secretory vesicles cannot be ruled out), with little if any mitochondrial localisation. In zygotes and ookinetes (stages when PbCAX is likely essential, as demonstrated here), PbCAX-GFP localisation was dispersed intracellularly across the parasite, with the majority not localised to mitochondria. At these stages a proportion of the observed PbCAX-GFP signal may associate with its production and trafficking, or with mislocalised protein, although if PbCAX-GFP was mislocalised during ookinete conversion then it would not allow development to proceed (in keeping with the knock-out results presented here).

PfCAX has a predicted mitochondrial targeting sequence at residues 11–18 (YVRRTISQ), consistent with mitochondrial localisation [7], and this is conserved throughout the apicomplexan CAXs. Interestingly, this protein sequence has been identified in phosphoproteomic studies [17], using preparations derived from mature trophozoite-infected erythrocytes, and two of these residues, T15 and S17, are putative phospho-acceptor sites (with ascores of 1000, suggesting the annotations have a high degree of confidence). Phosphorylation of the S17 residue of PbCAX has also been reported, in a similar study, using ookinete preparations (available on GeneDB). The homologous protein region of TgCAX was also identified in tachyzoite preparations, although it contained no phosphorylated sites [17]. However, neighbouring residues at positions S26, S27 and T46 were identified as being phosphorylated, albeit with lower ascores of 19, 13 and 6, respectively. Previous work has demonstrated that phosphorylation of the mitochondrial signal sequence of 2′,3′-cyclic nucleotide-3′-phosphodiesterase 2 (CNP2) alters its localisation so that it is retained in the cytoplasm [32] and its possible a similar mechanism could alter the location of apicomplexan CAXs. However, the localisation evidence presented here provides little evidence for mitochondrial function of CAXs in apicomplexan parasites.

### Roles and regulation of apicomplexan CAXs

Neither TgCAX nor PbCAX are required for asexually reproducing parasites. Most active Ca^2+^ transport pathways, including in apicomplexan parasites, play critical roles in providing Ca^2+^ tolerance, by refilling internal Ca^2+^ stores, and by shaping cytosolic free Ca^2+^ transients or oscillations that act as intracellular signals [33]. Previously identified Ca^2+^ efflux pathways include the plasma membrane and internally localised *P. falciparum* Ca^2+^-ATPase, PfATP4 [34], [35], the SERCA-type pumps at the endoplasmic reticulum in *P. falciparum* and *T. gondii*, PfATP6 and TgSERCA, respectively [5], [6], and the non SERCA-type *T. gondii* Ca^2+^-ATPase, TgA1, that localises to acidocalcisomes [36], [37]. Currently, the *in vivo* function of each Ca^2+^ efflux transporter (with respect to the cytosol) is unclear, although it is likely that there would be some overlap in the functions of these pathways. Thus, loss of one active Ca^2+^ transport mechanism might be compensated for by others, particularly during asexual development stages when the parasites are mainly intracellular, and do not face large extracellular Ca^2+^ concentrations [38]. In yeast and plants, Ca^2+^/H^+^ exchangers play major roles in providing tolerance to excess cytosolic Ca^2+^, due to their ability to function as low affinity, high capacity Ca^2+^ transporters. For example, in yeast the Ca^2+^/H^+^ exchanger Vcx1 efficiently sequesters Ca^2+^ into the vacuole when cytosolic Ca^2+^ levels are high without requiring the vacuolar Ca^2+^-ATPase [39]. However, deletion of vacuolar Ca^2+^/H^+^ exchange activity, such as in the *Arabidopsis cax1* knock-out, leads to up-regulation of vacuolar Ca^2+^-ATPase activity, as a compensatory mechanism [40] that may also manifest itself in apicomplexans.

Interestingly, PbCAX was essential during ookinete conversion, with knock-out lines failing to transform from zygotes and over an extended time period (in excess of 8 h) becoming necrotic or possibly apoptotic. Given that this phenotype could be reversed by the removal of extracellular Ca^2+^, this suggests that PbCAX's primary role is to provide a tolerance mechanism to environmental Ca^2+^ at a time when the parasite is extracellular and, at least initially, exposed to plasma concentrations of free Ca^2+^ of approximately 1.2 mM (it should be noted that our understanding of changes in the ionic environment in the mosquito midgut is very limited, presently). This is consistent with its primary role in plants and fungi [14].

Evidence is accumulating that Ca^2+^ exchangers play a role in directly regulating cytosolic Ca^2+^ elevations and in modulating cellular signaling associated with stress responses [33]. In the case of the sexual stages of *Plasmodium* parasites, it is well known that Ca^2+^ and Ca^2+^ interacting proteins regulate important parasite functions such as gametocyte differentiation into gametes, ookinete development and motility [41]–[45]. As PbCAX knock-out lines failed to transform from zygotes and long before becoming necrotic, it is tempting to speculate that PbCAX directly regulates the signaling pathways involved in zygote differentiation but further studies are required to answer this hypothesis.

Regulation of Ca^2+^/H^+^ exchangers, via protein modification or protein interaction, is consistent with their role as Ca^2+^ modulators. The *Arabidopsis* Ca^2+^/H^+^ exchanger AtCAX1 can be regulated via an autoinhibitory domain that is present on its long hydrophilic N-terminal tail [29]. Transport activity of AtCAX1 may be activated by phosphorylation or interaction with an activator protein at this domain [29], [46]. CrCAX1 from *Chlamydomonas* may also share this mechanism of regulation. This CAX protein has an extended hydrophilic N-terminal tail which when truncated leads to increased Ca^2+^/H^+^ exchange activity [10]. The CAX sequences from *P. falciparum* and other apicomplexan organisms likewise have a long N-terminal tail (Figure 1 and S1) and N-terminal truncation of PfCAX (to give sPfCAX) led to an increased Ca^2+^ tolerance phenotype in yeast (Figure 2) and enhanced Ca^2+^/H^+^ exchange activity (Figure 3). This may indicate that PfCAX (and by association other apicomplexan CAXs) has the potential to be regulated by an analogous mechanism. Phosphoproteomic data support this by suggesting the N-termini of PfCAX, PbCAX and TgCAX are phosphorylated ([17] and GeneDB).

The *S. cerevisiae* Ca^2+^/H^+^ exchanger Vcx1 does not have an extended N-terminal tail and is regulated by the Ca^2+^-dependent phosphatase calcineurin (PP2B) [11]. Functional calcineurin is absent in higher plants which may explain why regulatory mechanisms differ between plant and yeast CAX proteins, but a calcineurin phosphatase which requires Ca^2+^/calmodulin and is inhibited by immunosuppressant drugs (FK506 and cyclosporine A) is present in *P. falciparum* [47]. Some of the potential roles of calcineurin in *P. falciparum* are being elucidated [48], although its signaling roles and possible involvement in Ca^2+^ homeostasis including Ca^2+^ transporter regulation are unknown, but suggest alternative mechanisms of regulation and are worthy of investigation. Dysregulation of CAXs may prove equally as detrimental to plasmodial parasite survival at other life cycle stages, as PbCAX deletion does during ookinete conversion, and to other apicomplexans.

### Transmission blocking by targeting plasmodial CAXs

The data presented here confirm the functional Ca^2+^/H^+^ exchange activity of apicomplexan CAXs, shed light on possible CAX regulation, fail to support substantial mitochondrial localisation, demonstrate that CAXs are neither expressed to a high level nor required during asexual apicomplexan parasite development but PbCAX is expressed to a measureable level during sexual stages of development and is likely essential during ookinete conversion by protecting parasites against extracellular Ca^2+^.

In a new age of malaria eradication there is renewed interest in the development of transmission blocking therapies [49]. Most antimalarial treatments fail to kill circulating sexual parasite stages and thus do not stop transmission. Therefore, there is increasing interest in developing drugs and vaccines that can stop the transmission process. Here, PbCAX has been shown to be essential for parasite transmission. Furthermore, plasmodial CAXs are single copy genes with no close paralogues and CAXs are not found in higher animals. At present there are no potent and specific CAX inhibitors, KB-R7943 (a first-generation inhibitor of Na^2+^/Ca^2+^ exchangers) being the best known [7]. Our study has identified a new transmission blocking target, which is required for ookinete development by protecting the parasite from environmental Ca^2+^, and provides the experimental tools necessary to aid development of this therapeutic strategy.

## Materials and Methods

### Ethics statement

All animal work has passed an ethical review process and was approved by the United Kingdom Home Office. Work was carried out in accordance with the United Kingdom Animals (Scientific Procedures) Act 1986 and in compliance with European Directive 86/609/EEC for the protection of animals used for experimental purposes. All procedure were performed under Home Office licence number 40/3344.

### Apicomplexan CAX plasmid construction and yeast transformation

A synthetic version of *pfcax* cDNA was synthesised (Geneart) and codon optimised for expression in *S. cerevisiae* (Figure S3). The optimisation resulted in an increase in average GC content from 28% to 32%. This *pfcax* cDNA was sub-cloned into the *Xba*I and *Sac*I sites of the yeast expression vector piHGpd. N-terminal truncated *pfcax* and *TgCAX* variants (*spfcax* and *sTgCAX*), encoding proteins lacking the first 60 and 98 amino acids, respectively, were generated by PCR amplification using synthetic *pfcax* or *T. gondii* cDNA as template and the primers sPfCAXF/sTgCAXF and PfCAXR/TgCAXR (Table S1). The *spfcax* cDNA was cloned into the pGEM-T vector, while *sTgCAX* cDNA was cloned into the Strataclone vector for propagation and sequencing. *spfcax* was then sub-cloned into piHGpd, as above, while *sTgCAX* was subcloned into piUGpd. Plasmids were transformed into the *S. cerevisiae* strain K665 (*pmc1::TRP1 vcx1*Δ) [11], using the lithium acetate/polyethylene glycol method [50]. Transformed yeast colonies were grown in synthetic defined medium minus histidine or uracil and tryptophan for selective growth of the plasmid and maintenance of the insertional mutation. Expression of *pfcax* in yeast was confirmed by RT-PCR following extraction of yeast total RNA, using the acid phenol method [51]. Extracted RNA was further purified by phenol/chloroform/isoamyl alcohol extraction and isopropanol precipitation. First strand cDNA was produced from 1 µg of DNase-treated total RNA, using Superscript II reverse transcriptase (Invitrogen) and an oligo-dT/PfCAXR primer mix. PCR was performed using sPfCAXF/PfCAXR primers and yeast actin primers (Table S1), as a constitutive control. Transformation of *sTgCAX* in yeast was confirmed by PCR following extraction of genomic DNA. PCR was performed using the primers PUGF and PUGR (found approximately 200 base pairs either side of the multicloning site in piUGpd) and yeast actin primers (Table S1).

### Yeast assays

Ca^2+^ tolerance assays of K665 yeast expressing the synthetic *pfcax* and wild-type *sTgCAX* plasmids were performed on solid growth media and in liquid media. The previously characterised Ca^2+^/H^+^ exchanger sCrCAX1 [10] was used as a positive control for comparison with PfCAX and sPfCAX. Serial dilutions of yeast were grown at 30°C on solid YPD medium containing a 50 mM CaCl_2_ concentration. For determination of yeast growth rate in liquid media, yeast strains of the same starting cell density were inoculated in YPD medium containing a range of CaCl_2_ concentrations and grown at 30°C, shaking for 16 h in 24-well flat bottomed plates, and cell growth was determined by measuring absorbance at 600 nm.

Vacuolar membrane vesicles were isolated from yeast cells expressing *cax* plasmids, by purification of the microsomal fraction through a two-step sucrose gradient, as described previously [52]. By testing hydrolytic activity of the V-type H^+^-ATPase (V-ATPase) the isolated tonoplast fractions were all demonstrated to have no measurable contamination from other membrane fractions, as only the V-ATPase inhibitor bafilomycin (a tonoplast marker) inhibited H^+^-ATPase activity in these vesicles, while inhibitors of other membrane-localised H^+^-ATPases did not inhibit H^+^-ATPase activity (data not shown). Ca^2+^/H^+^ exchange activity was determined by measuring pH gradient-dependent ^45^Ca^2+^ uptake into membrane vesicles, as described previously [53].

### *P. berghei* culture and transfection

Transfection experiments were performed on *P. berghei* ANKA strain 2.34 parasites, as previously described [54]. The *pbcax* knock-out vectors were constructed for a double cross-over homologous recombination in the pBS-DHFR plasmid that contains a *Toxoplasma gondii dhfr/ts* cassette conferring resistance to pyrimethamine [55], [56]. The knock-out construct was generated by inserting 507 bp of the *pbcax* 5′ untranslated (UTR) region upstream and 497 bp of the *pbcax* 3′ UTR region downstream of the *dhfr* cassette (sequences of primers, N0431-4, used to amplify fragments from *P. berghei* genomic DNA are given in Table S1). The final knock-out construct was digested with *Apa*I and *Not*I to release the fragment for transfection. Transfection of *P. berghei* parasites with the knock-out construct was carried out in both wild-type parasites and in a line that constitutively expresses cytosolic GFP [18].

To generate a *pbcax-gfp* construct for a single cross-over homologous recombination, a 0.9 kb region of the *pbcax* gene without the stop codon was inserted in frame and upstream of the *gfp* sequence in the plasmid p277 containing the human *dhfr* cassette and conveying resistance to pyrimethamine [19]. Prior to transfection, the final construct was digested with *Pac*I. This cuts the plasmid in the middle of the insert, which is optimal for the homologous recombination event.

For the analysis of PbCAX-GFP localisation during the parasite's life cycle, images of GFP-expressing parasites were captured with a Zeiss AxioImager M2 (Carl Zeiss, Inc) microscope fitted with an AxioCam ICc1 digital camera (Carl Zeiss, Inc). Hoechst 33342 (Sigma) was used for nuclear staining of all stages and the P28 cy3-labelled antibody was used as a marker for female gametes/zygotes/ookinetes.

High resolution live cell imaging was performed using an Olympus-based personal Delta Vision work station at ×100 (numerical NA 1.4, oil). Subsequent off-line image preparation was carried out using Applied Precision software and finalised with Adobe Photoshop. Images presented are 2D projections of 0.1 µm stepped Z-stacks.

### *P. berghei* assays

For ookinete conversion assays, blood was taken by cardiac puncture from *P. berghei*-infected mice on day 4 post-infection into heparinised syringes, mixed with ookinete culture medium (RPMI1640 culture medium containing 25 mM HEPES, 25% (*v/v*) fetal bovine serum, 10 mM sodium bicarbonate, 50 µM xanthurenic acid, pH 7.6) and cultured at 19°C for a further 21–24 h before assessment of conversion. For direct immunolabelling (to aid identification), cultured cells were pelleted for 2 min at 800× *g* and then labelled for 10 min on ice in 50 µl of ookinete medium containing Hoechst 33342 and Cy3-conjugated mouse monoclonal antibody specific for P28 [21].

For exflagellation assays, mice were infected as described above. On day 4 to 5 post-infection, 10 µl of infectious tail snip blood was added to 40 µl of ookinete medium and incubated at room temperature for 15 mins. Gametocytaemia was 5–8%. The data were generated from the 15 min time point from 1 spot of tail blood performed in triplicate (from 3 different infected mice). An aliquot of 10 µl of tail blood was also analysed between 7 and 10 min in triplicate to ensure no exflagellation events were missed prior to microgametogenesis and in each case none were observed. Exflagellation was counted by measuring the number of exflagellation centres in 8 fields under magnification ×40 on a Zeiss Primostar microscope.

Parasite transmission to mosquitoes *in vivo* was assessed using mosquitoes fed directly on *P. berghei*-infected mice, as described previously [56]. Briefly, infected mice were offered to overnight-starved *A. stephensi* (SD 500 strain) mosquitoes in groups of approximately 100 for 30 min. Unfed mosquitoes were removed the next day, and the remaining mosquitoes were maintained at 19°C and 80% relative humidity on a 12-h light/dark cycle, being fed on a 2% (*w/v*) D-glucose solution that was replenished every 2 to 3 days. At 14 day post-feeding, mosquito midguts were dissected and analysed by fluorescence microscopy.

### *T. gondii* culture and transfection

*T. gondii* tachyzoites (RH*hxgprt*- and ΔKU80*hxgprt*- strains [24], [25]) were grown in confluent human foreskin fibroblasts (HFF) maintained in Dulbecco's Modified Eagle's Medium (DMEM, GIBCO, Invitrogen) supplemented with 10% (*v/v*) fetal calf serum, 2 mM glutamine and 25 µg/ml gentamicin.

Genomic DNA was prepared from tachyzoites (RH strain), using the Wizard SV genomic DNA purification system (Promega). Total RNA was isolated from tachyzoites, using Trizol (Invitrogen), and then total cDNA was generated by RT-PCR, using Superscript II reverse transcriptase (Invitrogen) according to the manufacturer's instructions. All amplifications were performed with LaTaq or ExTaq (TaKaRa) and the primers used are listed in Table S1. The full-length cDNA of *TgCAX* was amplified with primers pairs TgCAX-1/TgCAX-2, cloned into the pGEM-Teasy vector (Promega) and then sub-cloned into pTUB8MycGFPPfMyoAtailTy-HX [57] between the *Eco*RI and *Nsi*I sites to create the pTUB8TgCAX-Ty vector. For the knock-in vector, a fragment of genomic DNA corresponding to the C-terminal part of the gene was amplified using primers TgCAX-3/TgCAX-2 and cloned between *Kpn*I and *Nsi*I sites of the pTUB vector [57] modified to introduce 3 Ty-tags at the C-terminal end of the insert. This construct was then used to generate the knock-out vector by introducing a fragment of the 5′UTR of *TgCAX* amplified with primers TgCAX-4/TgCAX-5 and cloned between the *Bam*HI and *Not*I sites of the knock-in vector.

Parasite transfections were performed by electroporation as described previously [58]. The hypoxanthine-xanthine-guanine phosphoribosyl transferase (*hxgprt*) gene was used as a positive selectable marker in the presence of mycophenolic acid (25 mg/ml) and xanthine (50 mg/ml) as described before [59].

### *T. gondii* assays

For immunofluorescence assays (IFA), parasite-infected HFF cells were fixed with 4% (*v/v*) paraformaldehyde/0.05% (*v/v*) glutaraldehyde (PFA/GA) in phosphate buffered saline (PBS) and processed as described previously [60]. For generation of specific antibodies against TgCAX, the peptide NH_2_-GAPSRQLHLGLLSEGW-COOH was used to immunise two guinea pigs (Eurogentec) according to their standard protocol.

For plaque assays, host cells were infected with parasites for 7 days before fixation with PFA/GA. Giemsa staining was then performed as described previously [61].

For intracellular growth assays, HFF cells were inoculated with parasites and grown for 24 h before fixation with PFA/GA. IFAs were performed using α-gliding associated protein (GAP) 45 antibodies (a surface parasite marker). The number of parasites per vacuole was determined by counting the parasites in 100 vacuoles in duplicate in three independent experiments.

For egress assays, host cells were inoculated with freshly released parasites and grown for 30 h. Parasite-infected host cells were then incubated for 5 min at 37°C with DMEM containing 0.06% (*v/v*) dimethyl suphoxide (DMSO) or 3 µM of the Ca^2+^ ionophore A23187 from *Streptomyces chartreusensis* (Calbiochem) before fixation. IFAs were performed using α-GAP45 antibodies and the average number of egressed vacuoles was determined by counting 100 vacuoles for each condition in at least 2 independent experiments.

### Statistical analysis

Unpaired, two-tailed Student's *t*-tests or one-way analysis of variance (ANOVA) with Dunnett's post-hoc test were performed, as noted in the text, using the GraphPad Prism software program (version 5 for PC).

## Supporting Information

Figure S1 **Sequence alignments.** Amino acid sequence alignment of PfCAX with other apicomplexan CAX genes. Eight apicomplexan CAX sequences are shown. The Clustal W program was used to generate the alignment. The residues highlighted by a bold black line above correspond to transmembrane segment predictions determined with the TMHMM program (http://www.cbs.dtu.dk/services/TMHMM/). The residues highlighted by a bold green line below correspond to the conserved CAX regions, c-1 and c-2. Green shading denotes residues shown to be essential for Ca^2+^ transport in AtCAX1 and OsCAX1a [15], [16]. Yellow shading denotes the putative mitochondrial targeting motif [7]. Grey shading denotes cleaved sequences for mitochondrially imported proteins predicted by MitoProt II – v1.101 (http://ihg.gsf.de/ihg/mitoprot.html). Note that the MitoProt II probabilities for predicted mitochondrial targeting are 0.99, 0.98, 0.98, 0.93, 0.96, 0.98, 0.93 and 0.98 for PfCAX, PvCAX, PkCAX, PbCAX, TgCAX, NcCAX, CpCAX, EtCAX, respectively. Using TargetP 1.1 (http://www.cbs.dtu.dk/services/TargetP/) the probabilities are 0.74, 0.78, 0.81, 0.77, 0.89, 0.90, 0.90 and 0.92, respectively. Red shading denotes phospo-acceptor sites (GeneDB and [17]). CAX sequences are from (accession no.): Pf, *Plasmodium falciparum* (XP_966025.1); Pv, *Plasmodium vivax* (XP_001616060.1); Pk, *Plasmodium knowlesi* (XP_002261646.1); Pb, *Plasmodium berghei* (XP_678577.1); Tg, *Toxoplasma gondii* (XP_002369594.1); Nc, *Neospora caninum* (CBZ49795.1); Cp, *Cryptosporidium parvum*; Et, *Eimeria tenalla*. *Red letters*, identical or conserved residues in all sequences; *green letters*, conserved substitutions; *blue letters*, semi-conserved substitutions.
(DOC)

Figure S2 **Phylogenetic analysis.** Phylogenetic relationship of 14 CAXs between 12 members of the phylum *Apicomplexa* and 2 of the green algae *Chlamydomonas reinhardtii* at the amino acid level. CAX sequences are from (accession no.): Pf, *Plasmodium falciparum* (XP_966025.1); Pv, *Plasmodium vivax* (XP_001616060.1); Pk, *Plasmodium knowlesi* (XP_002261646.1); Pc, *Plasmodium chabaudi*; Py, *Plasmodium yoelii* (XP_725194.1); Pb, *Plasmodium berghei* (XP_678577.1); Tg, *Toxoplasma gondii* (XP_002369594.1); Nc, *Neospora caninum* (CBZ49795.1); Et, *Eimeria tenalla*; Cp, *Cryptosporidium parvum*; Ch, *Cryptosporidium hominis*; Cm, *Cryptosporidium muris* (XP_002142215.1); Cr; *Chlamydomonas reinhardtii* (CAR92574.1). The tree was generated using a ClustalW alignment of the full-length sequence using maximum likelihood under the WAG+F model of amino acid substitution, as implemented in RAxML v7.1 and using the fast bootstrap approach to determine tree confidence [62]. For bootstrapping, 100 iterations were performed. The tree was viewed using FigTree (tree.bio.ed.ac.uk/software/figtree). Bootstrap values are indicated at the nodes of branches. The branch length scale bar indicates the evolutionary distance of 0.2 amino acid substitutions per site.
(TIFF)

Figure S3 **Optimsed DNA sequence.** DNA sequence alignment of PfCAX cDNA compared with the yeast codon optimised synthetic PfCAX cDNA (synPfCAX).
(DOC)

Figure S4 **Ca^2+^ tolerance of yeast mediated by TgCAX.** (A) PCR analysis of *sTgCAX* transformation into yeast compared with yeast transformed with the empty vector control. (B) Saturated liquid cultures of K665 (*pmc1 vcx1*) yeast transformed with N-terminally truncated s*TgCAX* in piUGpd and empty vector alone were serially diluted to the cell densities as indicated, then spotted onto selection medium lacking uracil (SD –Ura) and YPD medium containing 50 mM CaCl_2_. Yeast growth at 30°C is shown after 3 days. A representative experiment is shown.
(TIF)

Figure S5 ***pbcax gfp*** **-tagging strategy and confirmation.** (A) Schematic representation of the gene targeting strategy used for tagging of the endogenous locus with *gfp* via single homologous recombination. Primers 1+2 (INT N43tag+ol492) used for diagnostic PCR are indicated. Probe location used for detection by pulse-field gel electrophoresis is indicated. (B) Diagnostic PCR confirming successful integration of the tagging sequence. Positive template controls (+ve control) amplifying a 517 bp region were performed using Control1 and Control2 primers. (C) Pulse-field gel electrophoresis blot hybridised with a probe to *hdhfr*, which detects the endogenous homologous locus on chromosome 7 and the disrupted locus on chromosome 1. (D) Western blot analysis using an anti-GFP antibody against control wild-type-GFP (wt) and transgenic (tag) activated gametocyte soluble and particulate fractions, showing bands of expected sizes of 29 kDa for wild-type-GFP and 77 kDa for PbCAX-GFP.
(TIF)

Figure S6 **PbCAX-GFP expression.** (A) Expression of PbCAX-GFP in live parasites at specific *P. berghei* life cycle stages is shown. Where appropriate, parasites are immunostained for the female gamete/zygote/ookinete marker P28 (red) and counterstained with the nuclear marker Hoechst 33342 (blue). GFP intensity is observed predominantly in female gameotyctes/female gametes/zygotes/ookinetes/oocysts and is far less prevalent in asexual blood stages/male gametocytes/male gametes. Scale bar: 5 µm. (B) High resolution deconvolution microscopy images of a live female gamete/zygote and an ookinete 24 h post activation expressing PbCAX-GFP and immunostained with P28 and counterstained with Hoechst 33342.
(TIF)

Figure S7 ***pbcax*** **disruption strategy and confirmation.** (A) Schematic representation of the gene targeting strategy used for gene disruption via double homologous recombination. Primers 1–4 (INT N43, ol248, N43 KO1 and N43 KO2) used for diagnostic PCR are indicated, as well as the *EcoR*I sites used for Southern blotting. Probe location used for detection by Southern blotting is indicated. (B) Diagnostic PCR confirming successful integration of the disruption sequence of *pbcax* in mutants N43 clone 9 (*cl9*) and N43-GFP clone 5 (*cl5 gfp*). Primers 1+2 (INT N43+ol248) were used to verify successful integration at the correct locus. Primers 3+4 (N43 KO1+N43 KO2) were used to confirm loss of the endogenous gene. (C) Southern blot analysis of *Eco*RI digested N43 clone 9 genomic DNA using the 5′ UTR of the targeting construct as a probe. Band sizes for N43 clone 9 (*cl9*) and wild-type (*wt*) are indicated. (D) Pulse-field gel electrophoresis blot hybridised with a probe to *tgdhfr/ts*, which detects the endogenous locus on chromosome 7 and the disrupted locus on chromosome 1.
(TIFF)

Figure S8 **Stability of** ***Δpbcax*** **parasites.** Bar graph illustrating the numbers of *Δpbcax cl9* parasites remaining in (ookinete) culture over time. As these parasite fail to convert into ookinetes, “round” form parasites were counted and their numbers presented per field of view (magnification, ×40; fields of view counted, 10). Bars represent the mean ± SEM of 3 repeats derived from cultured blood from a single infection.
(TIF)

Figure S9 ***TgCAX Ty*** **-tagging and disruption strategies and confirmation.** (A) Schematic representation of the *TgCAX Ty* transient transfection construct. (B) Immunofluorescence images of *Toxoplasma* tachyzoites stably transfected with *TgCAX Ty*, expressed under the control of the tubulin promoter. Note TgCAX-Ty (green) failed to colocalise with VP1 (red). (C) Schematic representation of the gene targeting strategy used for gene tagging the endogenous locus with *ty* via single homologous recombination. Primer set 1+2 (TgCAX-6+TgCAX-7) and 1+3 (TgCAX-6+P30A) used for diagnostic PCR are indicated. (D) Diagnostic PCR on cDNA confirming successful integration of the tagging sequence, expected sizes: 1+2: 843 bp and 1+3: 1008 bp. (E) Schematic representation of the strategy used for gene disruption via double homologous recombination. Primer sets 4+5 (TgCAX-8+TgCAX-2) and 4+6 (TgCAX-8+TgCAX-9) used for diagnostic PCR are indicated. (F) Diagnostic PCR on cDNA confirming successful gene deletion, expected sizes: 4+5: 1162 bp and 4+6: 695 bp. To confirm the presence of cDNA, two control genes were amplified GAP45 and profilin (PRF) with primers TgGAP45-1/2 and TgPRF-1/2, respectively. The expected sizes are 750 bp for GAP45 and 500 bp for PRF.
(TIF)

Protocol S1 **Genotype and Western blot analysis of** ***P. berghei*** **transfectants.**
(DOC)

Table S1 **Oligonucleotides used in this study.**
(DOC)
