# Supplementary material for: The Plasmodium berghei Ca2+/H+ Exchanger, PbCAX, Is Essential for Tolerance to Environmental Ca2+ during Sexual Development
Source: PLoS Pathog. 2013 Feb 28;9(2):e1003191. doi: 10.1371/journal.ppat.1003191 (PMC3585132; doi:10.1371/journal.ppat.1003191)
Supplement: Figure S1 — Sequence alignments. Amino acid sequence alignment of PfCAX with other apicomplexan CAX genes. Eight apicomplexan CAX sequences are shown. The Clustal W program was used to generate the alignment. The residues highlighted by a bold black line above correspond to transmembrane segment predictions determined with the TMHMM program (http://www.cbs.dtu.dk/services/TMHMM/). The residues highlighted by a bold green line below correspond to the conserved CAX regions, c-1 and c-2. Green shading denotes residues shown to be essential for Ca2+ transport in AtCAX1 and OsCAX1a [15], [16]. Yellow shading denotes the putative mitochondrial targeting motif [7]. Grey shading denotes cleaved sequences for mitochondrially imported proteins predicted by MitoProt II – v1.101 (http://ihg.gsf.de/ihg/mitoprot.html). Note that the MitoProt II probabilities for predicted mitochondrial targeting are 0.99, 0.98, 0.98, 0.93, 0.96, 0.98, 0.93 and 0.98 for PfCAX, PvCAX, PkCAX, PbCAX, TgCAX, NcCAX, CpCAX, EtCAX, respectively. Using TargetP 1.1 (http://www.cbs.dtu.dk/services/TargetP/) the probabilities are 0.74, 0.78, 0.81, 0.77, 0.89, 0.90, 0.90 and 0.92, respectively. Red shading denotes phospo-acceptor sites (GeneDB and [17]). CAX sequences are from (accession no.): Pf, Plasmodium falciparum (XP_966025.1); Pv, Plasmodium vivax (XP_001616060.1); Pk, Plasmodium knowlesi (XP_002261646.1); Pb, Plasmodium berghei (XP_678577.1); Tg, Toxoplasma gondii (XP_002369594.1); Nc, Neospora caninum (CBZ49795.1); Cp, Cryptosporidium parvum; Et, Eimeria tenalla. Red letters, identical or conserved residues in all sequences; green letters, conserved substitutions; blue letters, semi-conserved substitutions. (DOC) [file ppat.1003191.s001.doc]

PfCAX MVMGRVRATSYVRRTISQPLNKNV-------PPMKNMKNV-------------------NGLKDT 39

PvCAX MVMGRVRATSYVRRTISQPLNKNV-------APIKSSKST-------------------TGLNDT 39

PkCAX MVMGRVRATSYVRRTISQPLNKNV-------APIKNSQST-------------------TGLNDT 39

PbCAX MVMGRVRATSYIRRTISQPLNKN--------HPAGNIQNS-------------------RGLNDT 38

TgCAX MVMGRVRATSYVRRTLSQPVHKPRVSSVILPHPPQASVGSAGPGGTGSRGISVQLDDAVCHLTTG 65

NcCAX MVMGRVRATSYVRRTLSQPIHKPRVSSVILRQPPQPAAGS-GPGAAGGRDISAQLEDGVCHLTTG 64

CpCAX MVLQRMRATSYLRRTMSDPFSRRR-------TTLPYIVG-------------------------I 33

EtCAX MVMNRVRATSYVRRTLSQPMNKRRATAALAQHPDGSVIAS-------GG----DLQDSGGGILGS 54

PfCAX NLIRNRNLHLQLLCNNKMPAGM-------YDDELTKVYDLEETLP------FYYPRKSDIYGMQN 91

PvCAX NLIRNKNLHLQLLCNNKTPNGG-------VDDDLEQNYGYREIGA------YYSLKKSDVTGIYN 91

PkCAX NLIRNKNLHLQLLCNNKTPNGE-------LDDDLEQNFGYREIGA------YYSLRKSDITGLYN 91

PbCAX NLIGNKNLHLQLLCNGKEPYNE-------CIEHLGNYQDFDETKP------FYMPRKSDLNSIYS 90

TgCAX AGAPSRQLHLGLLSEGWNSLGGGEGRGSFLPSSADSRVIDEEAED--PNEEYRFTLKEDLQRIWS 128

NcCAX AGAPSRQLHLGLLSEGWSSLGG-AGEGAFMGPSSDSRVIDEEVED--LNDYYRFTLKEDMQRVWH 126

CpCAX FSQKN-DLCKNLMNTGISKNSF---------KETKIP---------------NYTWKSDAIAVLD 73

EtCAX VAAPNRQLHLGLLADGSSNSRL-------RDLGAEP--IEEETEGGAADARMDFSLKEDVARLKE 110

TM1

TM2

PfCAX MLNSKLNVLLIFVPIGLLSHFFGFKDIYIFFFNFMVLIPLSALMGHVTEDLALHTGEIIGGLLNA 156

PvCAX MLKNKLNIFLIFVPIGIISYLIGCKDIYIFFFNFMALIPLSALMGHVTEDLALHTGEIIGGLLNA 156

PkCAX MVKNKLNIFLIFVPIGIISHLIGCKDIYIFFFNFMALIPLSALMGHVTEDLALHTGEIIGGLLNA 156

PbCAX ILNNKLNILLLFIPIGIFSYLLEASDIYIFFFNFMALIPLSALMGNVTEDLALHTGEIIGGLLNA 155

TgCAX MLACRLNVLLFFVPLSFWSVVGSGSPLFVFICNFLALIPLASLLGNATEELALHTGEIIGGLLNA 193

NcCAX MLSGRLNLFLVFVPLSFWSVLVSGSPLFVFVCNFLALIPLASLLGNATEELALHTGEIIGGLLNA 191

CpCAX IFFTKLNILFLFVPFGVLSYYLEWGALATFWFNFIALIPLANLLGIFTEELALHTGEVVGGLLNA 138

EtCAX ICLTKTQLLLLFAPLGLWSRLGGLPTLCVFSFNFAALIPLSALLGAFTEELSLHTGEITGGLLNA 176

TM3

TM4

PfCAX TFGNLMEMIFSIQALNAGLINVVQGTLLGSILSNLLLVLGMSFFAGGLYHHIQKFNEKGATCSTS 221

PvCAX TFGNLMEMIFSIQALNAGLINVVQGTLLGSILSNLLLVLGMSFFAGGLYHHVQKFNEKGATCSTS 221

PkCAX TFGNLMEMIFSIQALNAGLINVVQGTLLGSILSNLLLVLGMSFFAGGLYHHVQKFNEKGATCSTS 221

PbCAX TFGNLMEMIFSIQALKAGLINVVQGTLLGSILSNLLLVLGMSFFAGGLYHHVQKFNEKGATCSTS 220

TgCAX TFGNAVEMIMSVQALRVGLLSVVQGTMLGSILSNLLLVLGMSFFAGGIRYHVQKFNEKGATCSVT 259

NcCAX TFGNAVEMIMSVQALRVGLLSVVQGTMLGSILSNLLLVLGMSFFAGGIRYHVQKFNEKGATCSVT 256

CpCAX TFGNAVEAILTVQGIRAGLITVVQGTLLGSILSNLLLVLGMSFFAGGIFHHVQKFNEKGASFSTS 203

EtCAX TFGNAVEMIMSVQALRIGLLEVVKGTLLGSILSNLLLVLGMSFFAGGLHHYLQKFNEKGATCSVT 240

**c1**

TM5

TM6

PfCAX LLLLSSLAITIPTVSSFTTNNNL--DVILKVSRITAVLIFVTYCLFLLFQLYTHISLFQDKEMTE 284

PvCAX LLLLSSLAITIPTVSSVTTNNNV--EVLLKVSRITAVLIFLTYCLFLLFQLYTHISLFQDKEMTE 284

PkCAX LLLLSSLAITIPTVSSVTTNNNV--EVLLKVSRITAVLIFLTYCLFLLFQLYTHISLFQDKEMTE 284

PbCAX LLLLSSLAITIPSVSSVTTNNNA--DVLLKVSRITAVLIFITYCLFLLFQLYTHISLFQDKEMTE 283

TgCAX LLLLSCMSIVIPTVAASGNDHANPTYDIIKISRTIAVLIGVTYCLFLFFQLYTHLNLFRDDEEGE 323

NcCAX LLLLSCMSIVIPTVAASGNDHASPTYDIIKISRTIAVLIGLTYCLFLFFQLYTHLNLFRDDEDGE 321

CpCAX LLMLSCMAISIPTIVAQFDLPQH---NILMISRLTAILLSFTYVLFLFFQLYTHINLFRDESVAS 265

EtCAX LLLLSCMGIVIPTVAAVDNGQHG-TYNILMISRITALLIGVTYCLFLFFQLYTHIGLFKDDDEDA 304

PfCAX E-----------------------------------------------------IPQLSVISGSI 296

PvCAX E-----------------------------------------------------VPQLSVLAGSI 296

PkCAX E-----------------------------------------------------EPQLSVLAGSI 296

PbCAX E-----------------------------------------------------TPQLSVITGSL 295

TgCAX EG----------------------------------------------------WPSMSWEAATV 336

NcCAX EE----------------------------------------------------WPSMSWEAATV 334

CpCAX NDKSYPSYMIDNISSVKYPNALPNIEIYNNNIYKNYPIISQNFNNYQLYDVCLELPTISWQIGTV 330

EtCAX EQ----------------------------------------------------WPMMSWEAATL 317

TM7

TM8

PfCAX FLILITLLVSIHSEFLIYSIDSVIKYYNISENFIGVILLPVVGNATEHLTAVTVAMKNKVDLTMG 361

PvCAX FLILITVLVSVHSEYLITTIEAVVKYYNISENFIGVILLPVVGNATEHLTAVTVAIKNKVDLTMG 361

PkCAX FLIIITILVSIHSEYLITTIEAVVKYYNISENFIGVILLPVVGNATEHLTAVTVAIKNKVDLTMG 361

PbCAX FLIIITFLVSVHSEYLINTVESVVRYYNISENFIGVILLPIVGNATEHLTAVTVAIKNKVDLTMG 360

TgCAX MLFIVTLLIAVHSEYLVGSIHDVVTNYGLPESFIGVILLPIVGNAAEHLTAVTVAMKNKVDLAMG 401

NcCAX ILFIVTLLIAVHSEYLVGSIHAVVTNYGLPESFIGVILLPIVGNAAEHLTAVTVAMKNKVDLAMG 399

CpCAX LILLCTILISIISECLVDSINGFISEWRFSENFIGVILLPLVGNAAEHITAVSVAIKNKTDLTIG 395

EtCAX MLFLVTSLVALHSELLVSSIEEVVADYGLSESFIGVILLPIVGNAAEHLTAVTVAMKNKVDLAMG

**c2**

TM9

TM10

PfCAX VAVGSSAQIALFVVPVTVLFGWILNKPMTLAFSPLSTVILVISVIVTMAIVQDGESNWLEGVLLI 426

PvCAX VAVGSSAQIALFVVPVTVLFGWILNKPMTLAFSPLSSVILVISVIVTMAIVQDGESNWLEGVLLI 426

PkCAX VAVGSSAQIALFVVPVTVLFGWILNQPMTLAFSPLSSVILVISVIVTMAIVQDGESNWLEGVLLI 426

PbCAX VAVGSSAQIALFVVPITVLFGWILNKPMTLAFSPLSSVILVISVIVTMAIVQDGESNWLEGVLLI 425

TgCAX VAVGSSAQIALFVFPFTVCAGWVLDQPLTLAVQPMNALVLLMAVLVAMAIVQDGESNWLEGVMLM 466

NcCAX VAVGSSAQIALFVFPFTVCAGWALDQPLTLAVQPMSALVLLMAVLVAMAIVQDGESNWLEGVMLM 464

CpCAX VAIGSSTQIALFVVPFSVIVGWLLGKPMTLSFTPVSAIILLLTNLIVIGIVQDGESNWFEGILLI 460

EtCAX VAVGSSAQIALFVFPFTVLVAWVMGQPLTMAVQPLSAVVMLLSVLVAMAVVQDGESNWLEGVMLM 382

TM11

PfCAX SAYLIVGVVFWFDTS---------------------- 441

PvCAX TAYLIVGVVFWFDTS---------------------- 441

PkCAX TAYLIVGVVFWFDTS---------------------- 441

PbCAX TAYLIVGVVFWFDGS---------------------- 440

TgCAX AAYLMIAIVFWYTDPNSSSGHSGPSVGKDTGVPGA-- 501

NcCAX AAYLMIAIVFWYNDPNSS-AHAATSVG-GKGAPASLP 499

CpCAX ISYCIVAVVYWYI------------------------ 473

EtCAX AAYLIISVVFWFDKPGPESAVSPASSA---------- 474
