# Supplementary figures and images for: The Plasmodium berghei Ca2+/H+ Exchanger, PbCAX, Is Essential for Tolerance to Environmental Ca2+ during Sexual Development
Source: PLoS Pathog. 2013 Feb 28;9(2):e1003191. doi: 10.1371/journal.ppat.1003191 (PMC3585132; doi:10.1371/journal.ppat.1003191)

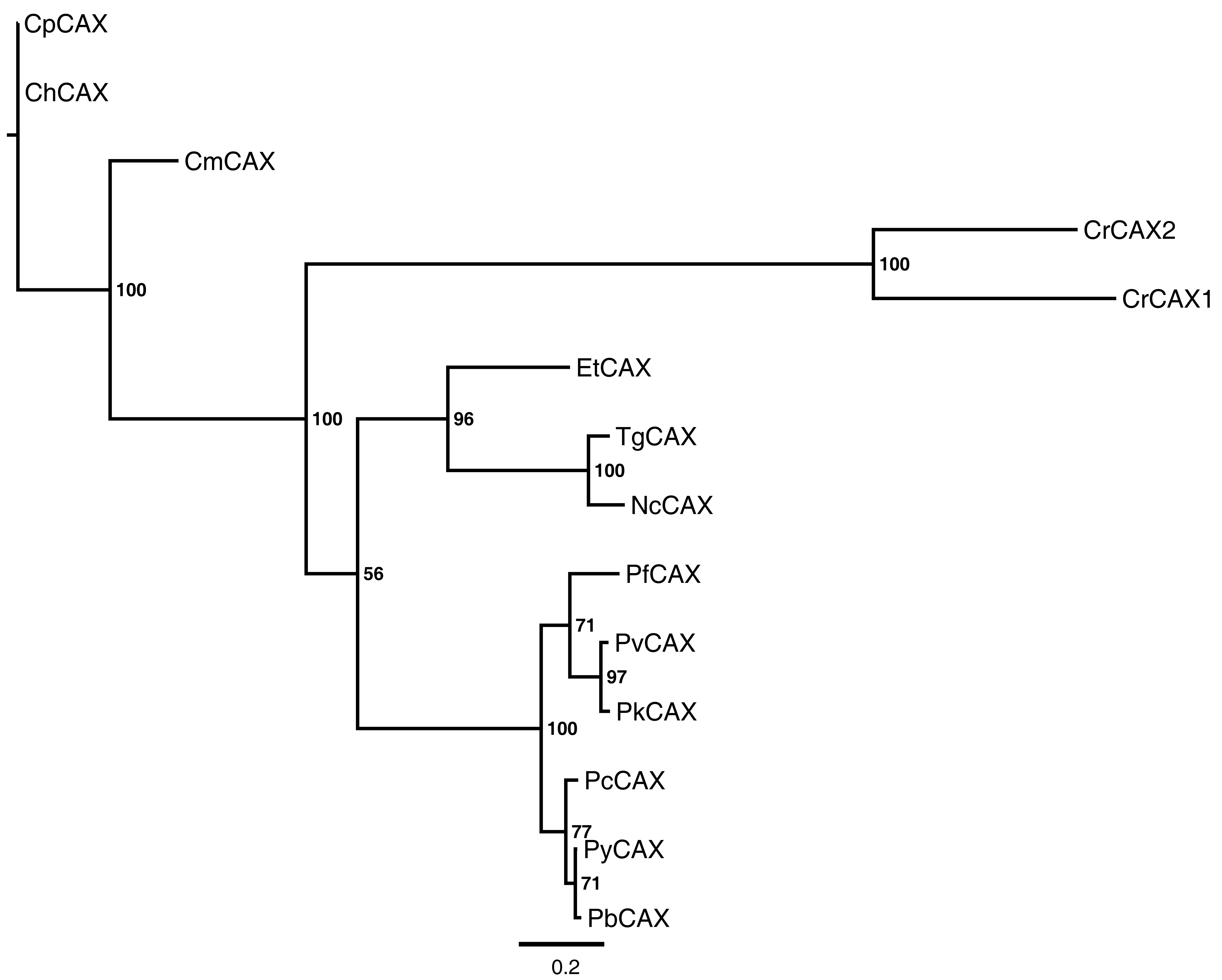

Supplement: Figure S2 — Phylogenetic analysis. Phylogenetic relationship of 14 CAXs between 12 members of the phylum Apicomplexa and 2 of the green algae Chlamydomonas reinhardtii at the amino acid level. CAX sequences are from (accession no.): Pf, Plasmodium falciparum (XP_966025.1); Pv, Plasmodium vivax (XP_001616060.1); Pk, Plasmodium knowlesi (XP_002261646.1); Pc, Plasmodium chabaudi; Py, Plasmodium yoelii (XP_725194.1); Pb, Plasmodium berghei (XP_678577.1); Tg, Toxoplasma gondii (XP_002369594.1); Nc, Neospora caninum (CBZ49795.1); Et, Eimeria tenalla; Cp, Cryptosporidium parvum; Ch, Cryptosporidium hominis; Cm, Cryptosporidium muris (XP_002142215.1); Cr; Chlamydomonas reinhardtii (CAR92574.1). The tree was generated using a ClustalW alignment of the full-length sequence using maximum likelihood under the WAG+F model of amino acid substitution, as implemented in RAxML v7.1 and using the fast bootstrap approach to determine tree confidence [62]. For bootstrapping, 100 iterations were performed. The tree was viewed using FigTree (tree.bio.ed.ac.uk/software/figtree). Bootstrap values are indicated at the nodes of branches. The branch length scale bar indicates the evolutionary distance of 0.2 amino acid substitutions per site. (TIFF) [file ppat.1003191.s002.tiff]

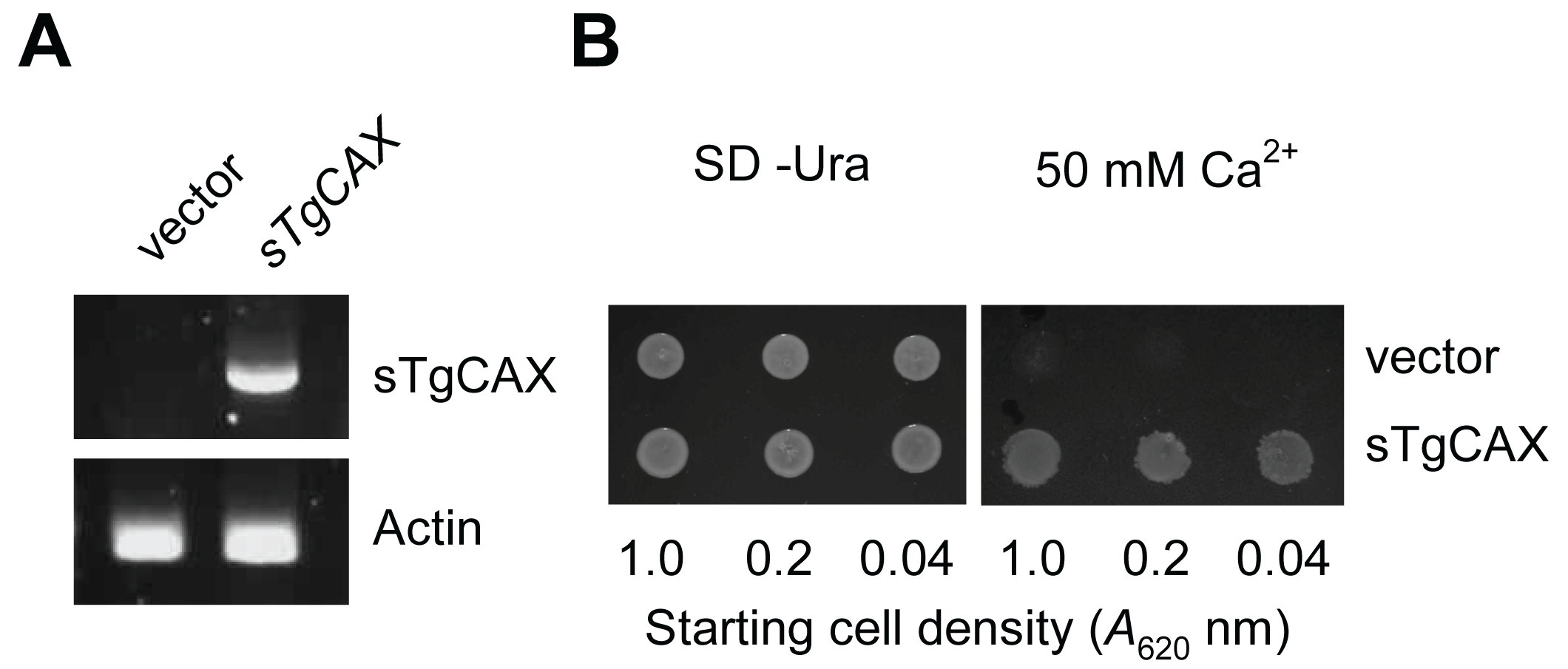

Supplement: Figure S4 — Ca2+ tolerance of yeast mediated by TgCAX. (A) PCR analysis of sTgCAX transformation into yeast compared with yeast transformed with the empty vector control. (B) Saturated liquid cultures of K665 (pmc1 vcx1) yeast transformed with N-terminally truncated sTgCAX in piUGpd and empty vector alone were serially diluted to the cell densities as indicated, then spotted onto selection medium lacking uracil (SD –Ura) and YPD medium containing 50 mM CaCl2. Yeast growth at 30°C is shown after 3 days. A representative experiment is shown. (TIF) [file ppat.1003191.s004.tif]

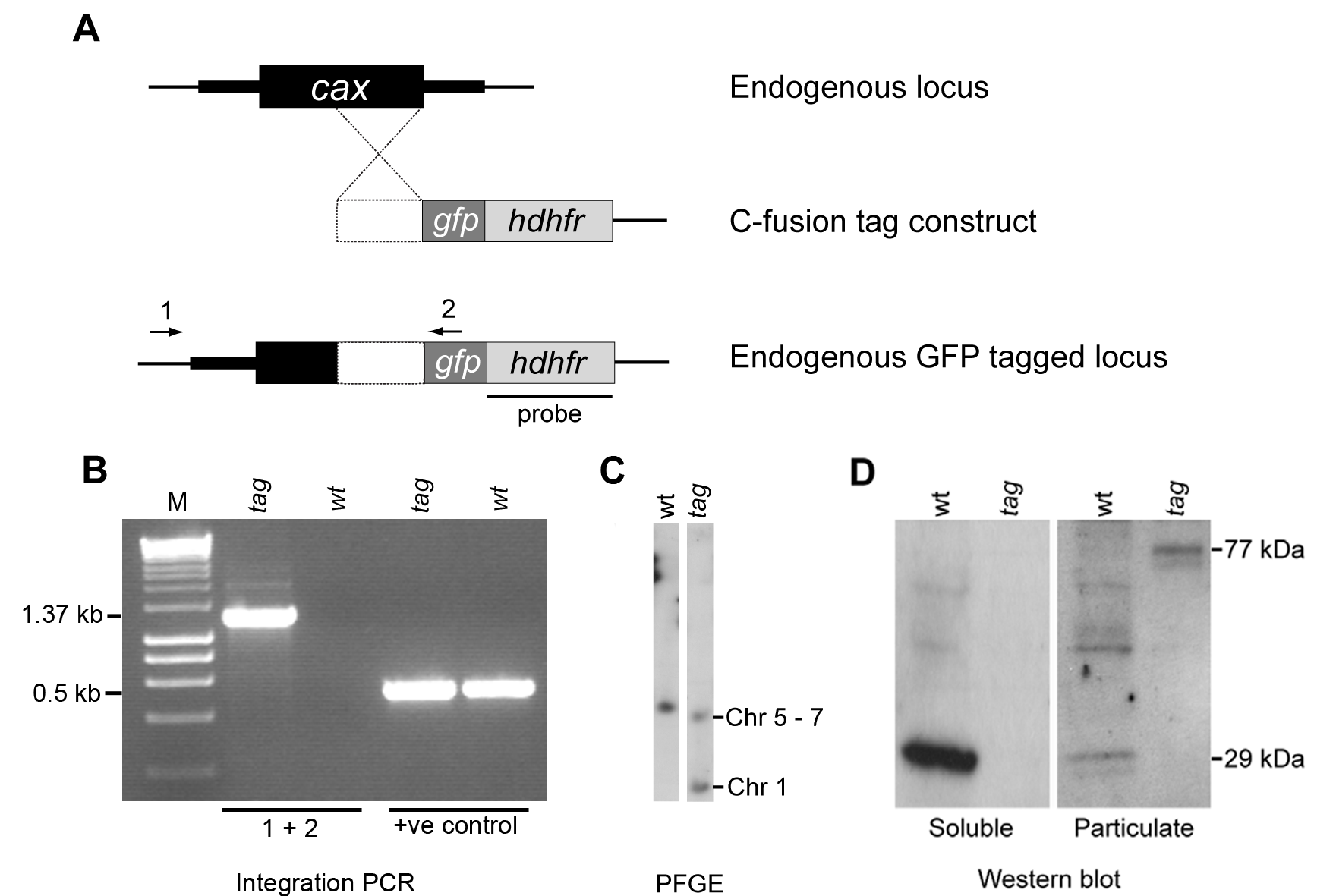

Supplement: Figure S5 — pbcax gfp -tagging strategy and confirmation. (A) Schematic representation of the gene targeting strategy used for tagging of the endogenous locus with gfp via single homologous recombination. Primers 1+2 (INT N43tag+ol492) used for diagnostic PCR are indicated. Probe location used for detection by pulse-field gel electrophoresis is indicated. (B) Diagnostic PCR confirming successful integration of the tagging sequence. Positive template controls (+ve control) amplifying a 517 bp region were performed using Control1 and Control2 primers. (C) Pulse-field gel electrophoresis blot hybridised with a probe to hdhfr, which detects the endogenous homologous locus on chromosome 7 and the disrupted locus on chromosome 1. (D) Western blot analysis using an anti-GFP antibody against control wild-type-GFP (wt) and transgenic (tag) activated gametocyte soluble and particulate fractions, showing bands of expected sizes of 29 kDa for wild-type-GFP and 77 kDa for PbCAX-GFP. (TIF) [file ppat.1003191.s005.tif]

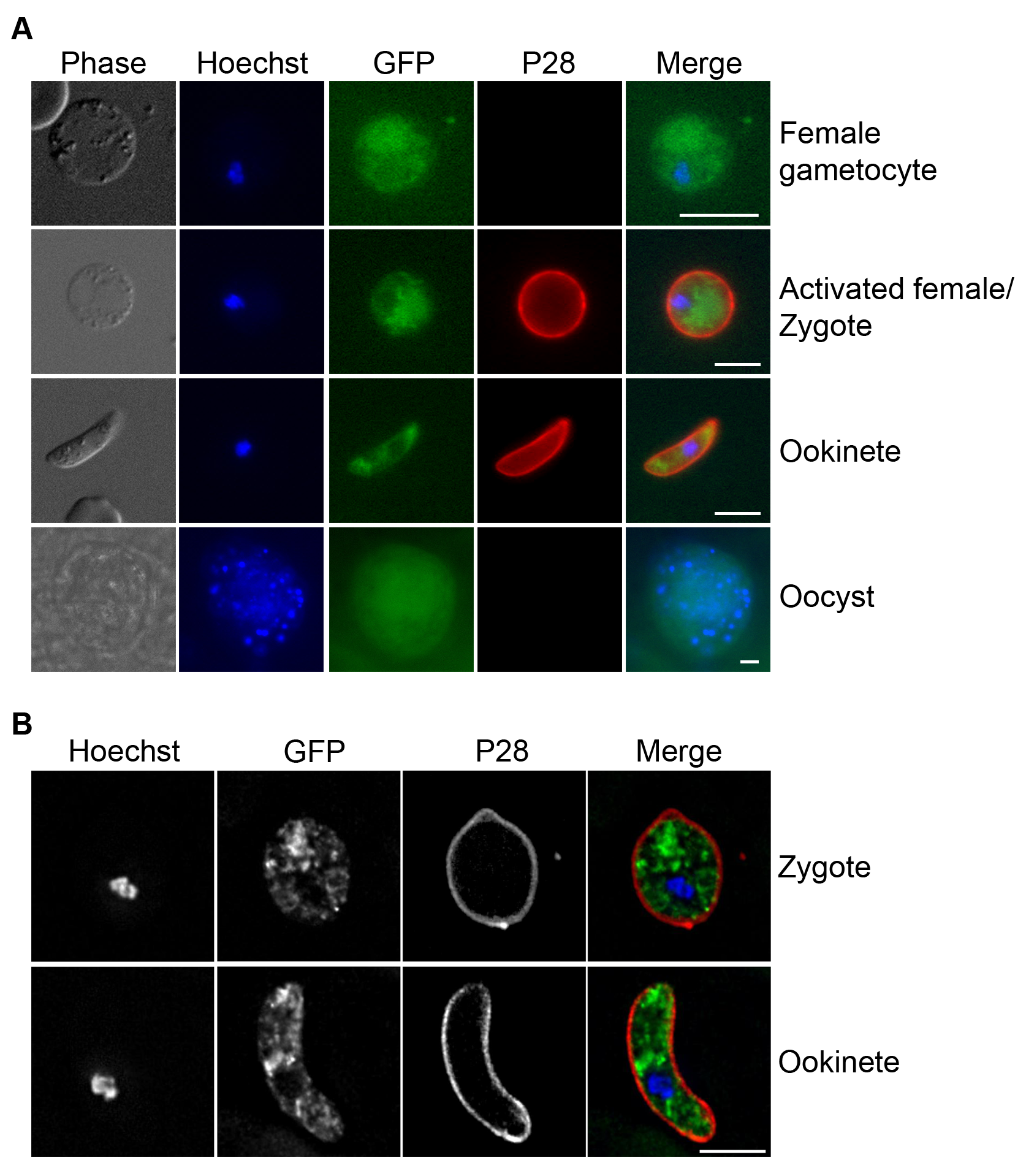

Supplement: Figure S6 — PbCAX-GFP expression. (A) Expression of PbCAX-GFP in live parasites at specific P. berghei life cycle stages is shown. Where appropriate, parasites are immunostained for the female gamete/zygote/ookinete marker P28 (red) and counterstained with the nuclear marker Hoechst 33342 (blue). GFP intensity is observed predominantly in female gameotyctes/female gametes/zygotes/ookinetes/oocysts and is far less prevalent in asexual blood stages/male gametocytes/male gametes. Scale bar: 5 µm. (B) High resolution deconvolution microscopy images of a live female gamete/zygote and an ookinete 24 h post activation expressing PbCAX-GFP and immunostained with P28 and counterstained with Hoechst 33342. (TIF) [file ppat.1003191.s006.tif]

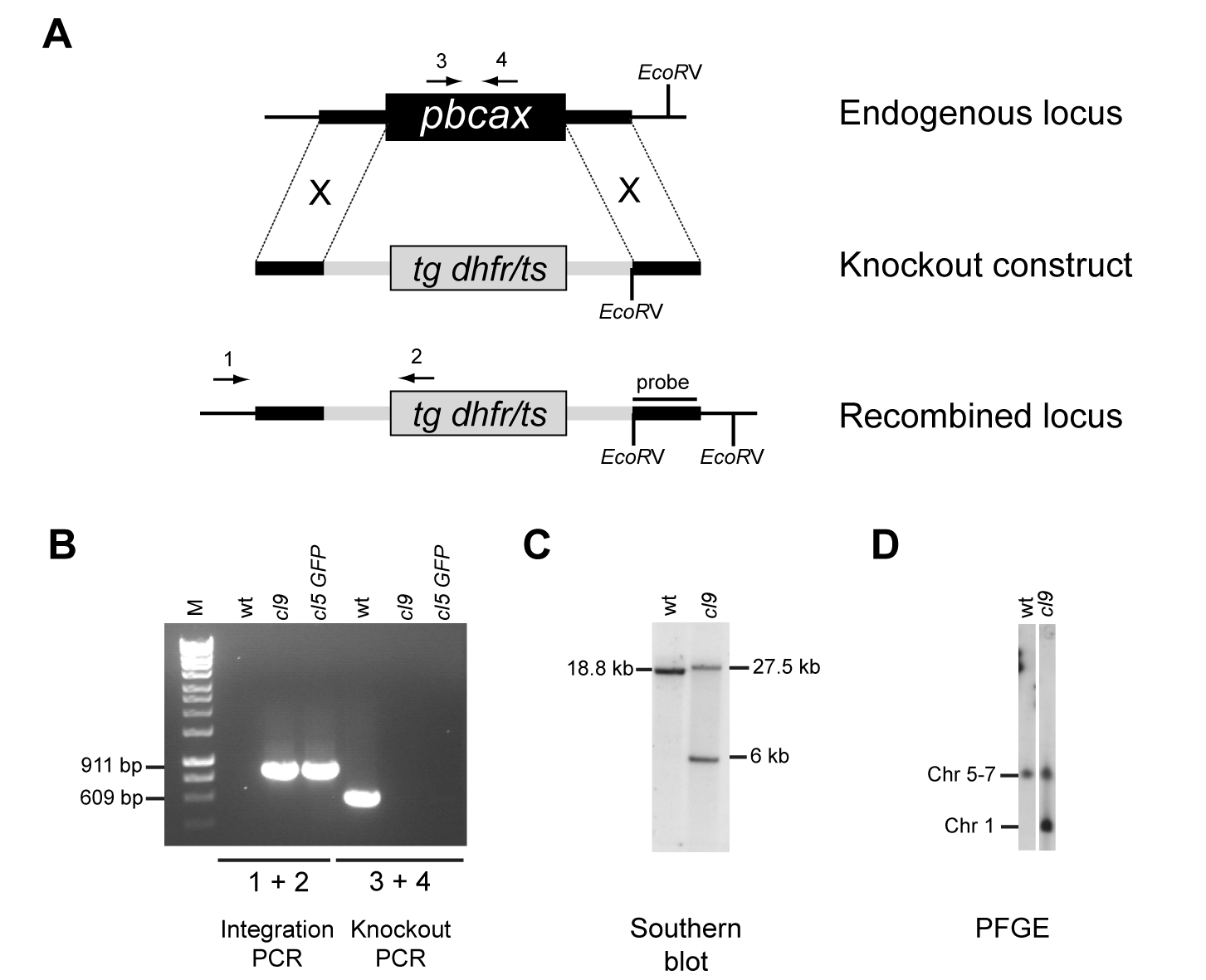

Supplement: Figure S7 — pbcax disruption strategy and confirmation. (A) Schematic representation of the gene targeting strategy used for gene disruption via double homologous recombination. Primers 1–4 (INT N43, ol248, N43 KO1 and N43 KO2) used for diagnostic PCR are indicated, as well as the EcoRI sites used for Southern blotting. Probe location used for detection by Southern blotting is indicated. (B) Diagnostic PCR confirming successful integration of the disruption sequence of pbcax in mutants N43 clone 9 (cl9) and N43-GFP clone 5 (cl5 gfp). Primers 1+2 (INT N43+ol248) were used to verify successful integration at the correct locus. Primers 3+4 (N43 KO1+N43 KO2) were used to confirm loss of the endogenous gene. (C) Southern blot analysis of EcoRI digested N43 clone 9 genomic DNA using the 5′ UTR of the targeting construct as a probe. Band sizes for N43 clone 9 (cl9) and wild-type (wt) are indicated. (D) Pulse-field gel electrophoresis blot hybridised with a probe to tgdhfr/ts, which detects the endogenous locus on chromosome 7 and the disrupted locus on chromosome 1. (TIFF) [file ppat.1003191.s007.tiff]

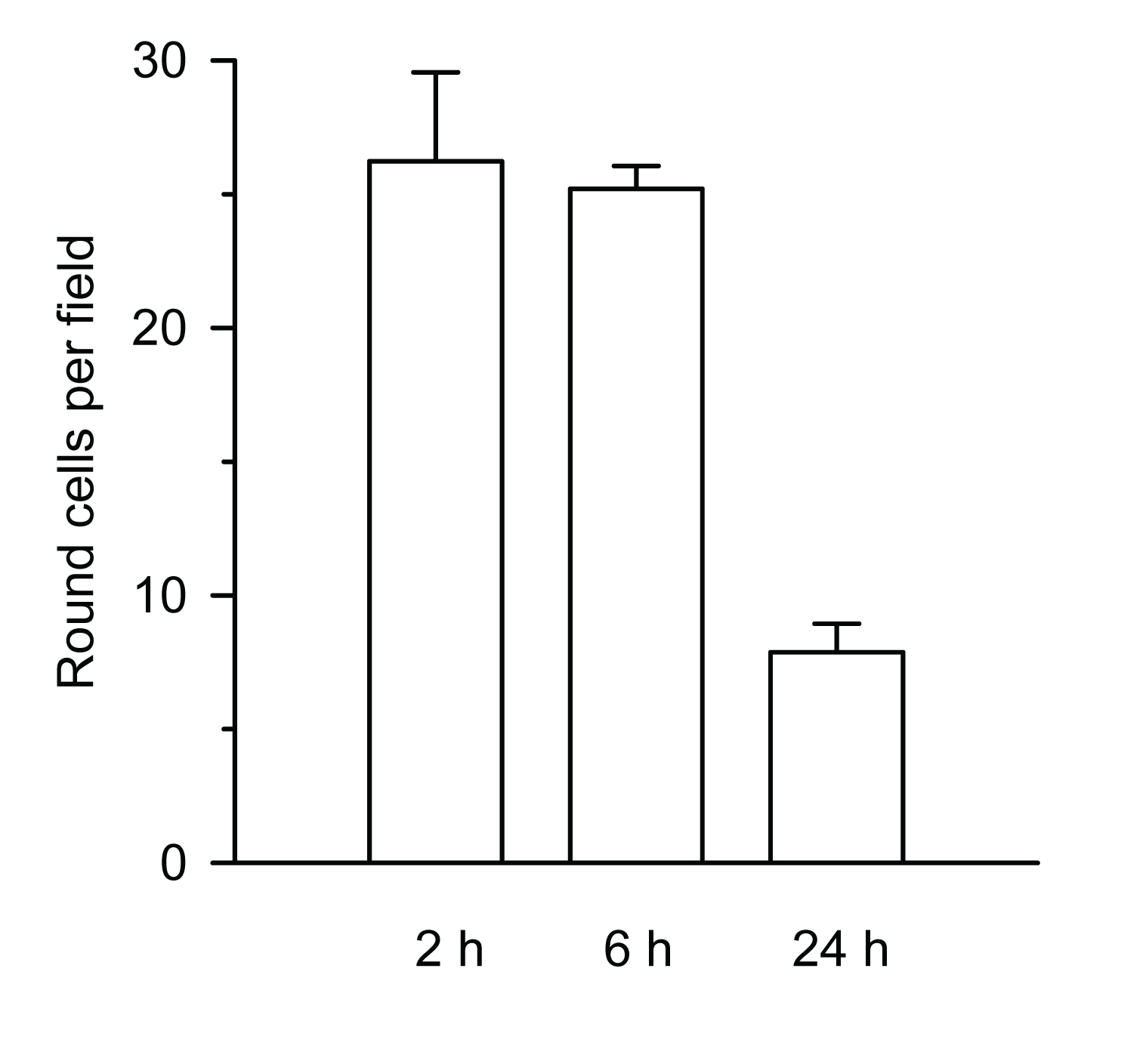

Supplement: Figure S8 — Stability of Δpbcax parasites. Bar graph illustrating the numbers of Δpbcax cl9 parasites remaining in (ookinete) culture over time. As these parasite fail to convert into ookinetes, “round” form parasites were counted and their numbers presented per field of view (magnification, ×40; fields of view counted, 10). Bars represent the mean ± SEM of 3 repeats derived from cultured blood from a single infection. (TIF) [file ppat.1003191.s008.tif]

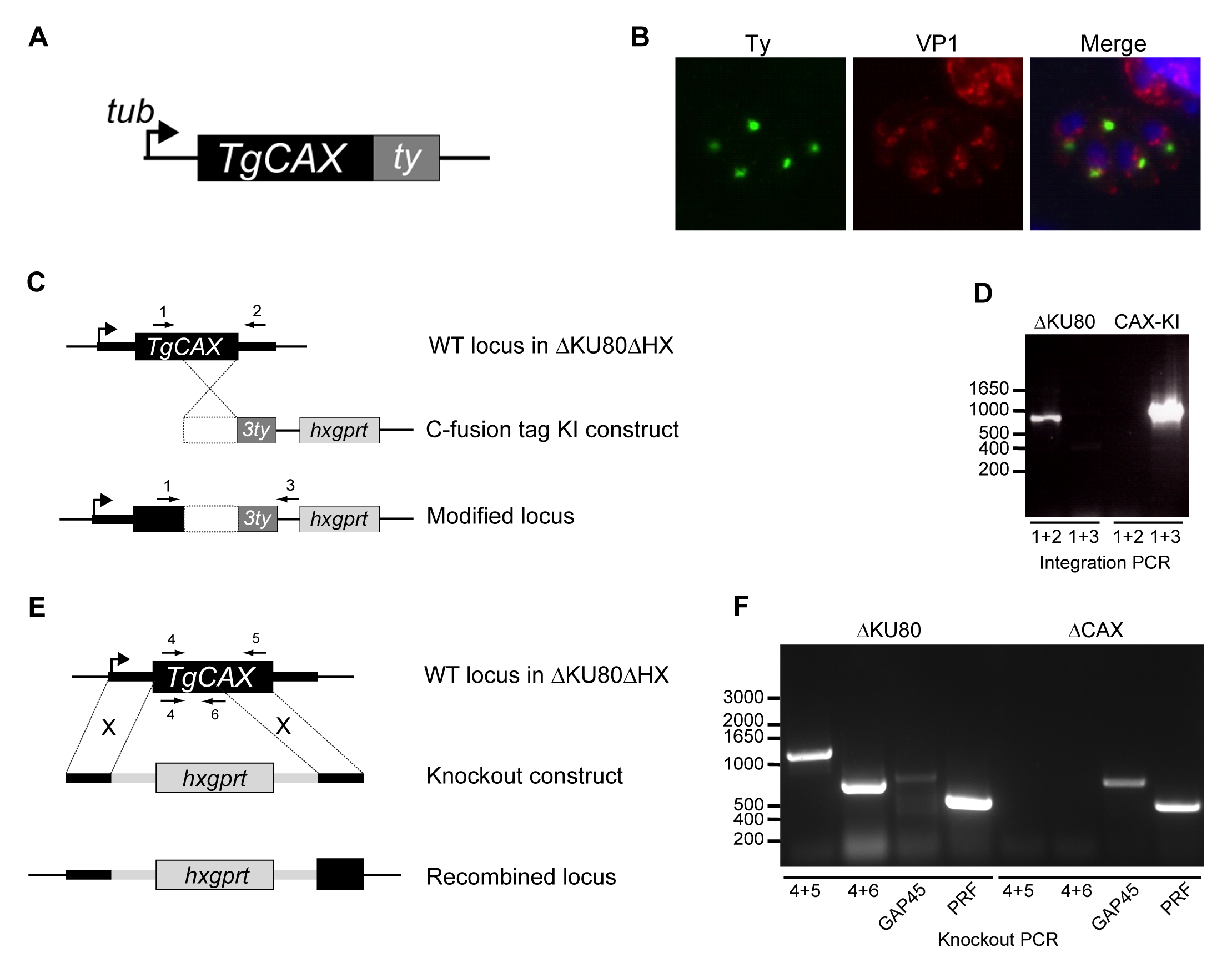

Supplement: Figure S9 — TgCAX Ty -tagging and disruption strategies and confirmation. (A) Schematic representation of the TgCAX Ty transient transfection construct. (B) Immunofluorescence images of Toxoplasma tachyzoites stably transfected with TgCAX Ty, expressed under the control of the tubulin promoter. Note TgCAX-Ty (green) failed to colocalise with VP1 (red). (C) Schematic representation of the gene targeting strategy used for gene tagging the endogenous locus with ty via single homologous recombination. Primer set 1+2 (TgCAX-6+TgCAX-7) and 1+3 (TgCAX-6+P30A) used for diagnostic PCR are indicated. (D) Diagnostic PCR on cDNA confirming successful integration of the tagging sequence, expected sizes: 1+2: 843 bp and 1+3: 1008 bp. (E) Schematic representation of the strategy used for gene disruption via double homologous recombination. Primer sets 4+5 (TgCAX-8+TgCAX-2) and 4+6 (TgCAX-8+TgCAX-9) used for diagnostic PCR are indicated. (F) Diagnostic PCR on cDNA confirming successful gene deletion, expected sizes: 4+5: 1162 bp and 4+6: 695 bp. To confirm the presence of cDNA, two control genes were amplified GAP45 and profilin (PRF) with primers TgGAP45-1/2 and TgPRF-1/2, respectively. The expected sizes are 750 bp for GAP45 and 500 bp for PRF. (TIF) [file ppat.1003191.s009.tif]
