# Supplementary material for: The Plasmodium berghei Ca2+/H+ Exchanger, PbCAX, Is Essential for Tolerance to Environmental Ca2+ during Sexual Development
Source: PLoS Pathog. 2013 Feb 28;9(2):e1003191. doi: 10.1371/journal.ppat.1003191 (PMC3585132; doi:10.1371/journal.ppat.1003191)
Supplement: Figure S3 — Optimsed DNA sequence. DNA sequence alignment of PfCAX cDNA compared with the yeast codon optimised synthetic PfCAX cDNA (synPfCAX). (DOC) [file ppat.1003191.s003.doc]

PfCAX 1 ATGGTTATGGGTAGAGTTCGTGCGACGTCTTATGTAAGGCGTACAATTTCACAACCGTTAAATAAAAATG
synPfCAX 1 ATGGTTATGGGTAGAGTTAGAGCTACTTCTTACGTTAGAAGAACTATTTCTCAACCATTGAACAAAAATG

PfCAX 71 TGCCCCCTATGAAAAATATGAAAAATGTAAACGGACTTAAGGATACCAATTTAATACGAAATAGAAATTT
synPfCAX 71 TTCCACCAATGAAGAATATGAAGAACGTTAATGGTTTGAAGGATACAAACTTGATTAGAAACAGAAACTT

PfCAX 141 ACATTTACAATTATTATGTAATAACAAAATGCCAGCAGGTATGTATGATGATGAATTAACAAAAGTGTAT
synPfCAX 141 GCATTTGCAATTGTTGTGTAACAACAAAATGCCTGCTGGCATGTATGATGATGAATTGACTAAGGTTTAC

PfCAX 211 GATTTAGAAGAAACGTTACCTTTTTATTATCCTAGAAAATCAGATATTTATGGAATGCAAAATATGTTAA
synPfCAX 211 GATTTGGAAGAAACTTTGCCATTTTACTACCCAAGAAAGTCTGATATCTACGGTATGCAAAACATGTTGA

PfCAX 281 ATAGTAAGTTAAATGTTTTATTAATATTTGTACCTATAGGATTATTAAGTCATTTCTTTGGTTTTAAAGA
synPfCAX 281 ACTCTAAGTTGAACGTTTTGTTGATTTTTGTTCCAATTGGTTTGTTGTCTCATTTTTTCGGTTTCAAGGA

PfCAX 351 TATATATATATTTTTTTTTAATTTTATGGTATTAATACCCTTATCTGCTCTTATGGGTCATGTAACTGAA
synPfCAX 351 TATCTACATTTTTTTCTTCAACTTCATGGTTTTGATTCCATTGTCTGCTTTGATGGGTCATGTTACTGAA

PfCAX 421 GATTTAGCATTACATACAGGAGAAATTATTGGAGGATTATTAAATGCTACGTTTGGTAATTTAATGGAAA
synPfCAX 421 GATTTGGCTTTGCATACTGGTGAAATTATTGGTGGTTTGTTGAATGCTACTTTTGGTAATTTGATGGAAA

PfCAX 491 TGATTTTTTCTATTCAAGCTTTGAATGCTGGATTAATAAATGTTGTTCAAGGTACTCTTCTTGGAAGTAT
synPfCAX 491 TGATTTTCTCTATTCAAGCTTTGAATGCTGGTTTGATTAATGTTGTTCAAGGTACTTTGTTGGGTTCTAT

PfCAX 561 CTTATCTAATTTACTTTTGGTTTTAGGTATGTCATTTTTTGCAGGAGGTTTATATCATCATATACAAAAA
synPfCAX 561 TTTGTCTAATTTGTTGTTGGTTTTGGGAATGTCTTTTTTTGCCGGTGGTTTGTATCATCATATTCAAAAG

PfCAX 631 TTTAATGAGAAAGGAGCAACATGTAGTACATCTCTTTTATTATTATCTAGTCTAGCTATAACTATACCAA
synPfCAX 631 TTCAATGAAAAAGGTGCTACTTGTTCTACTTCTTTGTTGTTGTTATCTTCTTTGGCTATTACTATTCCAA

PfCAX 701 CAGTATCATCATTTACAACTAATAATAATTTAGACGTTATCCTAAAGGTGTCAAGAATAACAGCTGTTTT
synPfCAX 701 CTGTTTCTTCTTTTACTACAAACAACAACTTGGATGTTATTTTGAAGGTTTCTAGAATTACTGCTGTTTT

PfCAX 771 AATATTTGTAACATATTGTTTATTTCTATTATTTCAATTATATACGCATATTTCTCTATTCCAAGATAAA
synPfCAX 771 GATTTTCGTTACTTACTGTTTGTTTTTGTTGTTTCAATTGTACACTCATATTTCTTTGTTCCAAGATAAA

PfCAX 841 GAAATGACTGAAGAAATTCCTCAATTATCTGTTATATCAGGATCGATCTTTTTAATATTAATTACTCTCT
synPfCAX 841 GAAATGACTGAAGAAATTCCACAATTGTCTGTTATTTCTGGTTCTATTTTCTTGATTTTGATTACATTGT

PfCAX 911 TGGTAAGTATACATTCAGAATTCCTTATTTATTCAATAGATTCAGTTATTAAATATTATAATATATCAGA
synPfCAX 911 TGGTTTCTATTCATTCTGAATTTTTGATTTACTCTATTGATTCTGTTATTAAGTACTACAACATTTCTGA

PfCAX 981 AAATTTTATAGGAGTTATACTTTTACCTGTTGTCGGTAATGCTACAGAACATTTAACAGCTGTTACTGTA
synPfCAX 981 AAACTTCATTGGTGTTATTTTGTTGCCAGTTGTTGGTAATGCTACTGAACATTTGACTGCTGTTACTGTT

PfCAX 1051 GCAATGAAAAATAAAGTTGATTTAACTATGGGAGTAGCTGTTGGATCTTCTGCACAAATAGCTCTTTTTG
synPfCAX 1051 GCTATGAAAAACAAGGTTGATTTGACTATGGGTGTTGCTGTTGGTTCTTCTGCTCAAATTGCTTTGTTTG

PfCAX 1121 TTGTACCTGTAACCGTATTATTTGGATGGATATTAAACAAACCCATGACTCTAGCCTTTTCCCCACTTTC
synPfCAX 1121 TTGTTCCAGTTACTGTTTTGTTTGGTTGGATTTTGAACAAACCAATGACTTTGGCTTTTTCTCCATTGTC

PfCAX 1191 TACTGTTATTCTAGTCATATCCGTTATTGTCACTATGGCTATTGTACAAGATGGAGAAAGCAATTGGTTA
synPfCAX 1191 TACTGTTATTTTGGTTATTTCTGTTATTGTTACTATGGCTATTGTTCAAGATGGTGAATCTAATTGGTTG

PfCAX 1261 GAAGGAGTTTTATTAATCTCAGCTTATCTTATTGTTGGTGTTGTTTTCTGGTTTGATACATCATAA
synPfCAX 1261 GAAGGTGTTTTGTTAATTTCTGCTTACTTGATTGTTGGTGTTGTTTTTTGGTTCGATACTTCTTAA
