# Supplementary material for: The Plasmodium berghei Ca2+/H+ Exchanger, PbCAX, Is Essential for Tolerance to Environmental Ca2+ during Sexual Development
Source: PLoS Pathog. 2013 Feb 28;9(2):e1003191. doi: 10.1371/journal.ppat.1003191 (PMC3585132; doi:10.1371/journal.ppat.1003191)
Supplement: Protocol S1 — Genotype and Western blot analysis of P. berghei transfectants. (DOC) [file ppat.1003191.s010.doc]

**Protocol S1.**

Genotype analysis of *P. berghei* transfectants

PCR, Southern blot and pulsed field gel electrophoresis (PFGE) analyses were used to determine whether the transfection construct had integrated into the correct locus in pyrimethamine-resistant parasites. For the C-fusion GFP tagged parasites, one diagnostic PCR reaction was used as illustrated in Figure S5A. Primers INT N43tag and ol492 (Table S1) were used to determine correct integration of the *gfp* sequence at the targeted locus. Primers Control1 and Control2 (Table S1) were used to verify the wild-type and tag DNA templates. For the gene deletion parasites, two diagnostic PCR reactions were used as illustrated in Figure S7A. Primers INT N43 and ol248 (Table S1) were used to determine correct integration of the selectable marker at the targeted locus. Primers N43 KO1 and N43 KO2 (Table S1) were used to verify successful deletion of the targeted gene.

Having confirmed integration, genomic DNA from wild-type and mutant parasites was digested with *EcoR*V and the fragments were separated on a 0.8% agarose gel, blotted onto a nylon membrane (GE Healthcare), and probed with a PCR fragment homologous to the *P. berghei* genomic DNA just outside of the targeted region.

Chromosomes of wild-type and gene deletion parasites were separated via pulsed field gel electrophoresis (PFGE) on a CHEF DR III (BioRad), using a linear ramp of 60–500 s for 72 h at 4 V/cm. Gels were blotted and hybridised with a probe recognising both the resistance cassette in the targeting vector and, more weakly, the 3’UTR of the *P. berghei dhfr/ts* locus on chromosome 7.

Western blot analysis *of P. berghei* transfectants

PbGFP_CON_ and *pbcax-gfp* activated gametocytes were prepared and purified, as described previously [1]. The resulting parasite pellets were resuspended in hypotonic buffer (10 mM TRIS HCl, pH 8.0, 5 mM EDTA), containing protease inhibitors (Roche), and centrifuged at 100,000 x *g* for 10 min at 4°C. The supernatants (soluble fraction) were saved and the pellets were then washed once and dissolved in the same buffer containing 500 mM NaCl and 1% (*v/v*) NP40, lysed at 4°C for 1 h and centrifuged at 100,000 x *g* for 1 h at 4°C. The resulting supernatants (particulate fraction) and the supernatants from the first centrifugation (soluble fraction) were then analysed by PAGE, and immunoblotting performed, using nitrocellulose membranes (Amersham Biosciences), anti-GFP rabbit polyclonal antibody (Invitrogen) and the Western Breeze Chemiluminescent Anti-Rabbit developing system (Invitrogen), according to the manufacturer’s instructions.

1. Guttery DS, Ferguson DJ, Poulin B, Xu Z, Straschil U, et al. (2012) A putative homologue of CDC20/CDH1 in the malaria parasite is essential for male gamete development. PLoS Pathog 8: e1002554.
