# Supplementary material for: The Plasmodium berghei Ca2+/H+ Exchanger, PbCAX, Is Essential for Tolerance to Environmental Ca2+ during Sexual Development
Source: PLoS Pathog. 2013 Feb 28;9(2):e1003191. doi: 10.1371/journal.ppat.1003191 (PMC3585132; doi:10.1371/journal.ppat.1003191)
Supplement: Table S1 — Oligonucleotides used in this study. (DOC) [file ppat.1003191.s011.doc]

**Table S1. Oligonucleotides used in this study**.

| Primer | Orientation | 5’- 3’ sequence |
| --- | --- | --- |
| sPfCAXF | sense | AAATCTAGAAAAATGTATGATGATGAATTGACTAAG |
| PfCAXR | antisense | AAAGAGCTCTTAAGAAGTATCGAACCAAAAAAC |
| sTgCAXF | sense | aaaTCTAGAAAAATGGCCGACTCTCGGGTCATCGAC |
| sTgCAXR | antisense | aaaGAGCTCTCAAGCCCCGGGCACTCCAGTG |
| ActinF | sense | CTTCTGCTCTCTCTGATTTG |
| ActinR | antisense | CATTCAGGCTGCGCAATG |
| PUGF | sense | CTTCTGCTCTCTCTGATTTG |
| PUGR | antisense | CATTCAGGCTGCGCAATG |
| TgCAX-1 | sense | aaaTCTAGAAAAATGGTGATGGGCCGCGTTCGC |
| TgCAX-2 | antisense | ggcATGCATTCCAGAAAACAATTGCAATCATCAGGTAGGC |
| TgCAX-3 | sense | ccgggtaCCTCCTCTGCTCCTGAAGGTCAC |
| TgCAX-4 | sense | CGGGATCCCTCGAGGCAGGAAACCCCACAGC |
| TgCAX-5 | antisense | GGCGCGGCCGCAGGGGAGGAAGAAGAGCCGCC |
| TgCAX-6 | sense | CCTGTCAGTCGTTCAAGGAACGATG |
| TgCAX-7 | antisense | GTGCTCAGAAACGCACGTCCG |
| TgCAX-8 | sense | AAATCTAGAAAAATGGCCGACTCTCGGGTCATCGAC |
| TgCAX-9 | antisense | ggccctgcaggCTCTTCGTCGTCTCGGAAAAGATTCAGG |
| P30A | antisense | CAGTTTCTTTATAATGGGGC |
| TgGAP45-1 | sense | GAATTCAAAATGGGAAACGCGTGCAAGAAGAAC |
| TgGAP45-2 | antisense | ATGCATCGTTCAACAAGGGTGCATCCGACAAGTCG |
| TgPRF-1 | sense | CCGGGATCCGACTGGGACCCTGTTGTCAAGG |
| TgPRF-2 | antisense | CCGTTAATTAATACCCAGACTGGTGAAGATACTCG |
| N0431 | sense | CCCCGGGCCCCAGTAATGATGTTCCCATAC |
| N0432 | antisense | GGGGAAGCTTCTCAATACATTCATTGTATGGCTC |
| N0433 | sense | CCCCGAATTCGCTAATTACTGCTTATCTTATTG |
| N0434 | antisense | GGGGTCTAGAGTAATAAATGTACTGTATGTG |
| T0841 | sense | CCCCGGTACCACATACAGGAGAAATTATTGGAGGGCTAC |
| T0842 | antisense | CCCCGGGCCCTGAACCATCGAACCAAAAAACAACACCAAC |
| INT N43tag | sense | AACCATCCTGCAGGGAATATACAA |
| Ol492 | antisense | ACGCTGAACTTGTGGCCG |
| Control1 | sense | CCCCGGGCCCCAAAGATCCATCAAAAAATAAAACAT |
| Control2 | antisense | GGGGAAGCTTCTATTAAAACAGGTAGTCATTTTGAG |
| INT N43 | sense | GCCTATGATATATATGCCTGCATGA |
| Ol248 | antisense | GATGTGTTATGTGATTAATTCATACAC |
| N43 KO1 | sense | CAAGCGCTAAAAGCAGGATT |
| N43 KO2 | antisense | AGCAATTTGAGCAGATGATCC |
